# Proteomic analysis of hepatic effects of phenobarbital in mice with humanized liver

**DOI:** 10.1007/s00204-022-03338-7

**Published:** 2022-07-26

**Authors:** Heike Sprenger, Josef Daniel Rasinger, Helen Hammer, Wael Naboulsi, Elke Zabinsky, Hannes Planatscher, Michael Schwarz, Oliver Poetz, Albert Braeuning

**Affiliations:** 1 Department of Food Safety, German Federal Institute for Risk Assessment, Max-Dohrn-Str. 8-10, 10589 Berlin, Germany; 2 Institute of Marine Research (IMR), Postboks 1870 Nordnes, 5817 Bergen, Norway; 3 SIGNATOPE GmbH, Markwiesenstr. 55, 72770 Reutlingen, Germany; 4 Department of Experimental and Clinical Pharmacology and Pharmogenomics, University of Tübingen, Wilhelmstr. 56, 72074 Tübingen, Germany; 5 NMI Natural and Medical Sciences Institute at the University of Tuebingen, Markwiesenstr. 55, 72770 Reutlingen, Germany

**Keywords:** Liver toxicity, Proteomics, Humanized mice, Phenobarbital, CAR activation

## Abstract

Activation of the constitutive androstane receptor (CAR) may induce adaptive but also adverse effects in rodent liver, including the induction of drug-metabolizing enzymes, transient hepatocellular proliferation, and promotion of liver tumor growth. Human relevance of CAR-related adverse hepatic effects is controversially debated. Here, we used the chimeric FRG-KO mouse model with livers largely repopulated by human hepatocytes, in order to study human hepatocytes and their response to treatment with the model CAR activator phenobarbital (PB) in vivo. Mice received an intraperitoneal injection with 50 mg/kg body weight PB or saline, and were sacrificed after 72–144 h. Non-repopulated FRG-KO mice were used as additional control. Comprehensive proteomics datasets were generated by merging data obtained by targeted as well as non-targeted proteomics approaches. For the first time, a novel proteomics workflow was established to comparatively analyze the effects of PB on human and murine proteins within one sample. Analysis of merged proteome data sets and bioinformatics data mining revealed comparable responses in murine and human hepatocytes with respect to nuclear receptor activation and induction of xenobiotic metabolism. By contrast, activation of MYC, a key regulator of proliferation, was predicted only for mouse but not human hepatocytes. Analyses of 5-bromo-2′-deoxyuridine incorporation confirmed this finding. In summary, this study for the first time presents a comprehensive proteomic analysis of CAR-dependent effects in human and mouse hepatocytes from humanized FRG-KO mice. The data support the hypothesis that PB does induce adaptive metabolic responses, but not hepatocellular proliferation in human hepatocytes in vivo.

**Supplementary Information:**

The online version contains supplementary material available at 10.1007/s00204-022-03338-7.

## Introduction

The constitutive androstane receptor (CAR), a xeno-sensing nuclear receptor, triggers pleiotropic responses in hepatocytes upon its activation by xenobiotic compounds. A plethora of chemicals are capable of activating CAR, for example environmental pollutants such as certain polychlorinated biphenyls (Gährs et al. 2013), or the antiepileptic drug phenobarbital (PB), which is often used as a model compound for CAR activation under experimental conditions (Kobayashi et al. 2015; Kodama and Negishi 2006). Clear species differences in CAR ligand binding have been described: for example, 1,4-bis-[2-(3,5-dichloropyridyloxy)]benzene (TCPOBOP) is an activator of murine but not human CAR (Ledda-Columbano et al. 2003), while 6-(4-chlorophenyl)imidazo (2,1-b) (1,3)thiazole-5-carbaldehyde O-(3,4-dichlorobenzyl)oxime acts agonistic at human but not murine CAR (Maglich et al. 2003). Additional species differences regarding CAR activation and related cellular consequences have been detected, e.g., for azole fungicides (Marx-Stoelting et al. 2017, 2020). In contrast to most known CAR activators, PB does not directly activate the receptor as a ligand, but appears to trigger CAR-dependent responses via indirect activation involving the epidermal growth factor receptor (Mutoh et al. 2013).

Observed hepatic consequences of CAR activation especially comprise liver hypertrophy, induction of a battery of drug-metabolizing enzymes such as cytochrome P450 (CYP) monooxygenases, transient hepatocellular proliferation, and non-genotoxic effects to promote liver tumorigenesis. For recent reviews on CAR activation and its consequences, please refer to Cai et al. (2021), Molnar et al. (2013), Wang et al. (2012), and Zhao et al. (2022). While CAR activation is often generally perceived as a mechanism of liver tumor promotion, observations in rodents indicate that both, tumor-inhibiting and -promoting effects may be observed, depending on the mouse model and treatment regimen chosen (e.g., see Braeuning et al. 2016; Lee 2000). Human relevance of liver tumorigenesis by PB and other activators of CAR is controversially discussed (Braeuning et al. 2015; Braeuning and Schwarz 2016; Elcombe et al. 2014; Yamada et al. 2015). While the activation of CAR in humans by PB is generally accepted, the fact that PB induces hepatocellular proliferation in mouse liver but not in cultured human hepatocytes in vitro (Haines et al. 2018; Parzefall et al. 1991; Plummer et al. 2019) has often been put forward as a key argument against human relevance of CAR-mediated non-genotoxic carcinogenesis in humans (Elcombe et al. 2014; Lake 2018; Yamada et al. 2021).

For a long time, analyses involving human hepatocytes have only been possible in vitro, harboring all inherent disadvantages, limitations, and difficulties with liver cell cultivation and in vitro-in vivo extrapolation (Godoy et al. 2013). Some years ago, novel chimeric mouse models have become available which allow for the analysis of the behavior of human hepatocytes in vivo in mouse livers repopulated with human hepatocytes, which have largely replaced the original population of mouse hepatocytes (Chow et al. 2016; Foquet et al. 2017; Katoh et al. 2008; Ohshita and Tateno 2017). Even if non-parenchymal cells are still murine in these models, they constitute, in terms of the degree of humanization, a remarkable improvement over mouse models bearing only individual human genes. One example for such a mouse model is the PXB mouse model (e.g., see Kakuni et al. (2013)). This model is based on uPA-SCID mice, providing immunodeficiency along with compromised hepatocyte function due to expression of the urokinase type plasminogen activator (uPA) transgene. Human hepatocytes are transplanted via the spleen and allow for a high degree of human hepatocyte repopulation. Another model is the FRG-KO mouse, where hepatocyte function is disturbed by loss of the *Fah* gene (encoding fumaryl acetoacetase), while immunodeficiency is due to a knockout of the *Rag2* and *Il2rg* genes (Azuma et al. 2007).

The PXB mouse model has been used for the yet only available short-term study of hepatocyte proliferation following activation of CAR by phenobarbital. In this study, Yamada et al. showed that hepatocellular proliferation did not occur in human hepatocytes (Yamada et al. 2014), supporting earlier findings in human hepatocytes in vitro (Braeuning and Schwarz 2016). More recently, samples from the above study have been used for a more comprehensive analysis of transcriptional responses, similarly showing a lack of proliferation of human hepatocytes following CAR activation (Yamada et al. 2020). However, available data on PB-induced changes in chimeric mice are still largely limited to the mRNA level, and artifacts caused by the individual background of a specific mouse model, as well as individual characteristics of the human hepatocyte donor, may limit the conclusiveness of a single study such as the above.

Therefore, we designed a study to monitor PB-induced hepatocellular effects in the FRG-KO chimeric mouse model. A broad proteomics approach comprising targeted and non-targeted analyses, together with extensive bioinformatic data evaluation, was followed to dissect the molecular consequences of PB treatment in human and murine cells.

## Materials and methods

### Animal experiment

The animal study was conducted at Yecuris (Tualaton, OR, USA). The previously described fumaryl acetoacetase (FAH)-deficient FRG-KO mouse model was used, allowing for the study of human hepatocytes in vivo in mouse livers repopulated with > 90% of human hepatocytes (Azuma et al. 2007). Adult female mice with a *Fah*^−/−^/*Rag2*^−/−^/*Il2rg*^−/−^ genotype (Yecuris, Tualaton, OR, USA) were used. These mice were either kept on nitisinone to compensate for their genetic metabolic deficiency plus additional prophylactic trimethoprim/sulfamethoxazole antibiotics treatment (in the following termed FRG-KO, possessing only their original hepatocytes), or were repopulated with human hepatocytes (humanized mice, termed hu-FRG-KO,  > 90% repopulation rate as checked by human albumin production). Human hepatocytes from a 13-year-old female Caucasian donor (Yecuris, Tualaton, OR, USA; lot no. HHF13023) were used for liver repopulation.

**Fig. 1:**
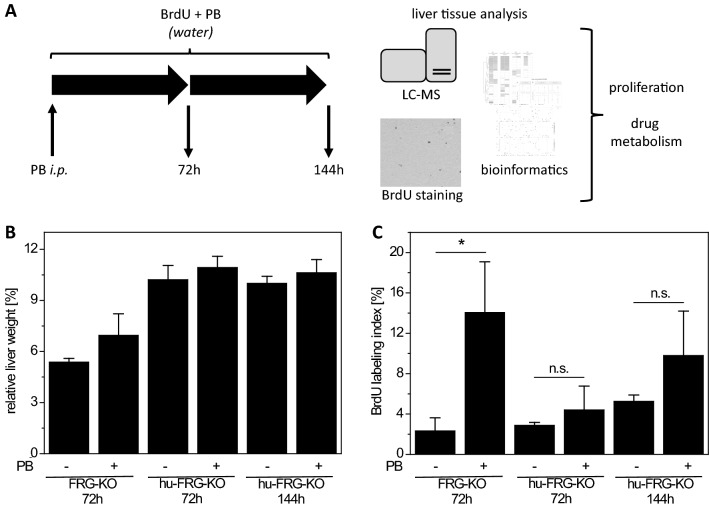
Analysis of PB effects in humanized liver mice. **A** Schematic representation of experimental workflow. **B** Relative liver weights (expressed as percent of body weight) and **C** BrdU incorporation (expressed as percentage of BrdU-positive hepatocyte nuclei) for FRG-KO mice (without human hepatocyte repopulation) and hu-FRG-KO mice (repopulated with human hepatocytes) after administration of PB for 72 h and 144 h. Mean ± SD (*n* = 3–4 mice per condition) are shown. Statistical significance: *p < 0.05; *n.s.* not significant

Mice were treated intraperitoneally with a single injection of 50 mg/kg body weight PB (dissolved in 0.9% saline), or with 0.9% saline as solvent control. Subsequently, the mice received tap water supplemented with 0.8 mg/mL 5-bromo-2'-deoxyuridine (BrdU) and 3% dextrose; the water for the PB treatment groups additionally contained 0.1% (w/v) PB. BrdU- and PB-containing water was freshly prepared each day. For an illustration of the experimental protocol, please refer to Fig. 1A. A similar protocol for PB and BrdU treatment has been used previously, showing extremely pronounced induction of proliferation and drug-metabolizing enzymes following injection of 90 mg/kg body weight PB, followed by 72 h on PB-supplemented water (Braeuning et al. 2011a). Dosing from the latter study was adapted for the present experiment by reduction of the initial dose to account for a possibly lowered tolerance towards PB in the humanized mice. Ethical approval for the animal experiment (no. DN000024) was obtained from the Institutional Animal Care and Use Committee at Yecuris.

Animals were inspected daily for clinical signs of toxicity and body weight was recorded. Sample collection was performed after 72 h or 144 h following anaesthesia with a combination of ketamine, xylazine and acepromazine. Livers were excised, weighed, cut, and samples were either snap-frozen and stored at −80 °C for further analysis, or fixed in Carnoy’s fixative for immunostaining as previously described (Braeuning et al. 2011b). Table 1 provides an overview of animals and treatment groups (a complete list of individual animals and respective phenotypic data can be found in Supplemental Table S1; a statistical summary in Supplemental Table S2).

**Table 1:** Overview of treatment groups of the animal experiment

Group	Mouse type	Donor	Treatment	Harvest at 72 h	Harvest at 144 h
A	hu-FRG-KO	HHF13023 (humanized)	0.9% Saline	4 Mice	4 Mice
B	hu-FRG-KO	HHF13023 (humanized)	50 mg/kg b.w. PB	4 Mice	4 Mice
C	FRG-KO	NA (no donor)	0.9% saline	3 Mice	
D	FRG-KO	NA (no donor)	50 mg/kg b.w. PB	3 Mice	

### Immunostaining

Double staining for FAH and BrdU incorporation was performed using sequential staining for FAH and BrdU incorporation on 5 µm thick slices of formalin-fixed tissue. To this end, an antibody directed against FAH (1:1.000 dilution; catalog no. 20-0034; Yecuris, Tualaton, OR, USA) was used in combination with a biotinylated anti-rabbit antibody (1:200, catalog no. 111-065-003; Dianova, Hamburg, Germany) and streptavidin-conjugated alkaline phosphatase (1:200, catalog no. 016-050-084; Dianova, Hamburg, Germany) using FastRed (Kementec, Taastrup, Denmark) as a substrate. Subsequent BrdU staining was performed as previously described using an anti-BrdU antibody (1:100; catalog no. M0744; Agilent/Dako, Santa Clara, CA, USA) together with a peroxidase-conjugated anti-mouse secondary antibody (1:100; catalog no. A2554; Sigma, Taufkirchen, Germany) and diaminobenzidine as a substrate. Slices were counterstained with hematoxylin. For analysis of BrdU incorporation, representative images of stained slices were acquired and at least 750 cells per animal were counted to determine the BrdU labeling index (i.e., the percentage of BrdU-positive nuclei).

### Targeted immunoaffinity proteomics 

CYP enzymes and transporter proteins were quantified by targeted immunoaffinity proteomics as described previously (Hammer et al. 2020; Wegler et al. 2017). In brief, frozen liver tissue (~ 10 mg) was grinded using a ball mill (Micro-dismembrator S, Sartorius, Göttingen). The powder was incubated for 1 h with lysis buffer and the resulting protein concentration was determined by BCA assay (Thermo Fisher Scientific, Waltham, MA, USA). Subsequently, samples were proteolyzed with trypsin overnight. Proteins were analyzed in several multiplex assays using 10–40 µg protein per analysis. Endogenous and stable isotope-labeled peptides were precipitated by TXP antibodies (SIGNATOPE, Reutlingen, Germany) using protein G-coated magnetic beads (Thermo Fisher Scientific, Waltham, MA, USA). The precipitated peptides were subsequently quantified using previously described 10 min and 20 min LC–MS methods (UltiMate 3000 RSLCnano and tSIM–QExactive Plus; Thermo Fisher Scientific, Waltham, MA, USA). Raw data were processed using the Skyline software (MACOSS Lab, Department of Genome Sciences, University of Washington, Seattle, USA). Peak areas of isotope-labeled peptides representing known peptide amounts and endogenous signals were set in relation to one another at the parent ion level. Analyzed proteins are listed with uniprot ID and surrogate peptide used for the quantification in Supplemental Table S3 (respective raw data in Supplemental Table S4).

### Non-targeted immunoaffinity proteomics

Sample preparation including tissue lysis and protein digestion was performed as for targeted immunoaffinity proteomics analysis (see above). Analyses were carried out at Signatope (Reutlingen, Germany).

#### LC–MS/MS analysis

The TXP immunoaffinity enrichment approach was chosen as previously outlined (Planatscher et al. 2010; Poetz et al. 2009). A theoretical target liver protein list was generated via protein atlas (v. 19). C-terminal-anchored alignment was then performed for all peptide sequences derived from in silico tryptic digest of target liver proteins to prioritize the selection of available TXP antibodies. Eventually, the specificity and functionality of 60 TXP antibodies were tested in tryptically digested hepatocyte lysates. Finally, the top 10 TXP antibodies which provided the best complementary liver protein coverage were then selected for this analysis.

60 µg proteolyzed liver tissue lysate was mixed with the 10 TXP antibodies (1 µg per antibody) in a 96-well microtiter plate. The immunoprecipitation procedure was performed using protein G-coated magnetic beads (Thermo Fisher Scientific, Waltham, MA, USA). After eluting peptides into 20 µl 1% formic acid, one quarter of the peptides were pre-concentrated for 2 min on the trap column Acclaim (0.3 mm I.D. × 5 mm, 5 µm, Thermo Fisher Scientific, Waltham, MA, USA) at a flow rate of 20 µL min^−1^ (2% acetonitrile and 0.05% trifluoroacetic acid). The peptides were then separated on an analytical column Acclaim PepMap RSLC C18 (75 µm I.D. × 150 mm, 2 µm, Thermo Fisher Scientific, Waltham, MA, USA) over 10 min using a gradient ranging from 4 to 55% buffer B (1% formic acid, 80% acetonitrile) at a flow rate of 300 nL min^−1^ at 40 °C. All spectra and ion chromatograms were recorded on a Q Exactive Plus Orbitrap Mass Spectrometer (MS; Thermo Fisher Scientific, Waltham, MA, USA) equipped with a nano-spray ion source in positive electrospray mode. The MS was operated in a data-dependent mode. Full MS spectra were acquired in the profile mode at resolution of 70,000. The mass range was set as 300–2000 m/z. The ten most abundant precursor ions in the MS scan were selected for collision-induced dissociation (CID) fragmentation and MS/MS analysis at resolution of 17,500. Normalized collision energy (CE) was set at 27. The mass isolation window for precursor was set at 2 m/z and dynamic exclusion was 5 s.

#### Protein identification and quantification

The generated raw data from the LC–MS/MS analysis were imported to Progenesis QI software (ver. 2.0.5387.52102, Nonlinear Dynamics Ltd., Newcastle upon Tyne, UK) for label-free quantification. The retention time (RT) of eluted peptides was aligned to a reference run to correct any potential RT shift. For feature selection, only ions with charges from + 2 to + 5 and more than two isotopic peaks were included. The raw abundances of the selected features were normalized based on total protein amount, a built-in method in Progenesis QI. MS/MS spectra were then exported as pepXML file format, and then imported in Proteome Discoverer (version 2.1, Thermo Fisher Scientific, Waltham, MA, USA). Peptide ions were searched via SEQUEST in mouse (UP000000589_10090) or human (UP0000005640_ 9606) UniProt database. SEQUEST search parameters were set as follows: 10 ppm precursor mass tolerance, 0.02 Da fragment mass tolerance, trypsin as enzyme; one missed cleavage site allowed, oxidation (H, M and W) and carbamidomethyl (C) were set as dynamic and static modifications, respectively. The percolator implemented in Proteome Discoverer was used to calculate the false discovery rate (FDR) of the identified peptides and only peptides with FDR < 1% were considered. Human and mouse search results deriving from the Proteome Discoverer were imported into Progenesis QI and analyzed separately. Protein abundance was extrapolated from the sum of unique normalized peptide ion abundances corresponding to that protein. Only non-conflicting peptides were considered for the relative quantification.

### Proteomics by shotgun mass spectrometry

#### LC–MS/MS analysis

Samples were prepared for untargeted proteomic analysis as previously described (Bernhard et al. 2018) following protocols and procedures at the Proteomics Unit at the University of Bergen (PROBE), Norway. In short, tissue protein was extracted and solubilized in lysis buffer (4% SDS, 0.1 M Tris–HCl, pH 7.6). Following sonication (Q55 Sonicator, Qsonica, CT, USA) and centrifugation (10 min at 13,000 rpm), supernatants were collected, and protein concentrations were determined (Pierce™ BCA Protein assay kit; Thermo Fisher Scientific, Waltham, MA, USA). Protein extracts were trypsin digested following a Filter Aided Sample Preparation (FASP) digestion protocol (Wisniewski et al. 2009). Tryptic peptides (0.5–1 μg) dissolved in 2% acetonitrile and 0.1% formic acid were injected into an Ultimate 3000 RSLC system (Thermo Fisher Scientific, Waltham, MA, USA) connected to a linear quadrupole ion trap-orbitrap (LTQ-Orbitrap Elite) mass spectrometer (Thermo Fisher Scientific, Waltham, MA, USA) equipped with a nanospray Flex ion source (Thermo Fisher Scientific, Waltham, MA, USA).

#### Protein identification and quantification

Raw data obtained in data-dependent acquisition (DDA) mode were analyzed as described by Tyanova et al. (2016). In short, MaxQuant (Cox and Mann 2008) with the built-in search engine Andromeda (Cox et al. 2011) was used for protein identification and protein quantification. MaxQuant (version 1.6.4.0) parameter settings were set as described before (Bernhard et al. 2018). Only reviewed protein sequences of both mouse (Uniprot proteome: UP000000589, accession date: 14.11.2019) and human (Uniprot proteome: UP000005640, accession date: 14.11.2019) reference proteomes were used for protein identification. False discovery rates (FDR) for peptide and protein identification were set to 1%; only unique peptides were used for label-free quantification (LFQ).

### Bioinformatics and statistical analysis

#### Immunoaffinity proteomics

The detected peptides were classified as species-specific or unspecific and multiple peptides per protein were summarized. Technical replicates were summarized and normalized protein abundance data generated by Progenesis QI were log_2_-transformed for further data analysis.

#### Shotgun mass spectrometry

Quality control of MaxQuant results was performed by the PTXQC pipeline (version 0.92.6) in R (Bielow et al. 2016). Processing of MaxQuant output was done by proteus R package (version 0.2.14) which included filtering out contaminants (Gierlinski 2018). Peptide and protein tables were created from evidence data by makePeptideTable and makeProteinTable, respectively, retaining only proteins identified by at least two peptides. Moreover, proteins with an extremely large number of missing values (> 90%) across all samples were removed. One biological replicate (mouse ID 64,246, sample ABE-7, hu-FRG-KO, PB treatment, 72 h; Supplemental Table S1) that behaved as an extreme outlier, with substantially more murine and less human proteins than the other humanized animals (Supp. Figure 12), was not included in the data set. Data were normalized to median intensity per sample and log_2_-transformed for further data analysis.

Statistical analyses were conducted in R version 4.0.3 (R Core Team 2020). Comparisons between saline- and PB-treated mouse samples were performed using a *t*-test and results were considered significant at *p* values below 0.05. Heatmaps were generated by the R package ComplexHeatmap version 2.6.2 (Gu et al. 2016) using default settings if not mentioned otherwise. Intersections of gene sets were visualized as UpSet plots using R package UpSetR version 1.4.0 (Convay 2019). Principal component analysis (PCA) was applied on scaled and centered data by the R-package pcaMethods (Stacklies et al. 2007).

#### Ingenuity pathway analysis

Data were analyzed with QIAGEN’s Ingenuity Pathway Analysis (IPA, QIAGEN Redwood City, CA, USA; www.qiagen.com/ingenuity; release 2014-06-24). We performed an upstream regulator analysis to determine transcription regulators potentially activated or inhibited. The right-tailed Fisher’s exact test was used to estimate the probability of association of a set of genes in the dataset with a transcription regulator by random chance alone. A *p*-value < 0.05 was chosen as significance level. Results were filtered to include only ligand-dependent nuclear receptors and transcription regulators. The *p*-value was used to evaluate the statistical significance of the overlap between the dataset genes and the genes that are regulated by the transcription regulator. The IPA z-score algorithm was used to identify transcription regulators that are expected to be activated or inhibited. A z-score ≥ 2 or ≤ −2 predicts a significantly activated or inhibited transcription regulator state, respectively.

#### Ortholog mapping

Orthologs were retrieved using the HCOP database (Eyre et al. 2007) aggregating information from 12 resources (bulk download from https://www.genenames.org/tools/hcop/ on 19 May, 2021). Uniprot IDs were joined via HGNC ID and MGI ID for human and mouse proteins, respectively, and filtered for entries with at least three orthology sources. For this purpose, tab-separated protein annotation files from UniProtKB were downloaded (on 19 May, 2021) including HGNC IDs and MGI IDs.

## Results

### General observations

Central aim of the study was to comparatively analyze effects of PB treatment on human and mouse hepatocytes in vivo in humanized and control mice by proteomics approaches. Different proteomics approaches may yield complementary results, and it has been demonstrated that targeted and non-targeted data sets can be combined to substantially extend their value for bioinformatic data evaluation (Kling et al. 2022). Therefore, we decided to use both, non-targeted and targeted approaches, to maximize proteomic information. In total, three different types of analysis, i.e., shotgun MS, non-targeted IA-MS, and targeted IA-MS were performed. Crucial hepatic effects of PB treatment, including the induction of DME and proliferation, have been reported to occur during the first 72 h after treatment (e.g. see Braeuning et al. (2011b)). Therefore, hu-FRG-KO mice were treated for 72 h and additionally for 144 h, to cover also the possibility that responses of human hepatocytes occur in a delayed manner. FRG-KO mice without repopulation were used as a positive control after 72 h of treatment (see Fig. 1A and overview in Table 1).

Animals did not show clinical signs of toxicity or pronounced weight changes during the experiment in daily visual inspections (Supplemental Table S1). Relative liver weights were increased by PB in FRG-KO mice without human hepatocyte repopulation, even though the difference did not reach our criteria for statistical significance (Fig. 1B; cp. Supplemental Table S2). Relative liver weights of the hu-FRG-KO mice were generally much higher than in their non-humanized counterparts, and were not strongly affected by PB (Fig. 1B). The CAR activator PB significantly induced proliferation in murine hepatocytes in FRG-KO mice (*p* = 0.02; Fig. 1C). A tendency for increased proliferation after administration of PB for 72 h or 144 h was observed, but failed the pre-defined criteria of statistical significance (*p* = 0.24 and *p* = 0.08 for 72 h and 144 h, respectively; Fig. 1C). Taken together, these results suggest that PB had clearly induced a proliferative response in mouse hepatocytes, whereas the response, if present at all, had been considerably less pronounced in human hepatocytes.

### Overview of proteomics data

To dissect molecular consequences of PB treatment in human and murine hepatocytes, three different proteomics datasets were acquired. Three complementary methods were used to maximize the informative value of generated data: a shotgun proteomics approach was followed to cover the entire proteome, while a non-targeted immunoaffinity proteomics (IA-Ms) approach was applied in parallel focusing on the hepatocyte proteome. In addition, targeted IA-MS was performed to specifically analyze the abundance of xenobiotic-metabolizing enzymes and transporters, as known targets of PB in the liver. The complete non-targeted immunoaffinity proteomics data set comprised 1032 peptides corresponding to 819 unique proteins (Supplemental Table S5). After filtering for species-specific peptides and summarizing multiple peptides per protein, we obtained 150 human and 220 mouse proteins. The unfiltered shotgun MS data set included 45,014 peptides corresponding to 6616 unique proteins. The filtered set covered 1298 human and 1615 mouse proteins based on species-specific and protein-specific peptides (Supplemental Table S6). The targeted IA-MS approach was tailored to detect CYPs and transport proteins related to xenobiotic metabolism, a major target of PB in liver cells, and was performed for a total of 42 unique proteins (Supplemental Table S4).

As illustrated in Supplemental Figure S1, the majority of proteins detected by non-targeted IA-MS proteomics (approx. 65–75%) were also detected by shotgun MS. Interestingly, proteins detected exclusively by one method tended to have a lower abundance compared to commonly detected proteins (Supplemental Figure S2). That is true for proteins specific for non-targeted IA-MS (Supplemental Figure S2A–B), as well as for proteins detected only by shotgun MS (Supplemental Figure S2C–D). This indicates that both methods are complementary and deliver unique information in their protein sets.

**Fig. 2:**
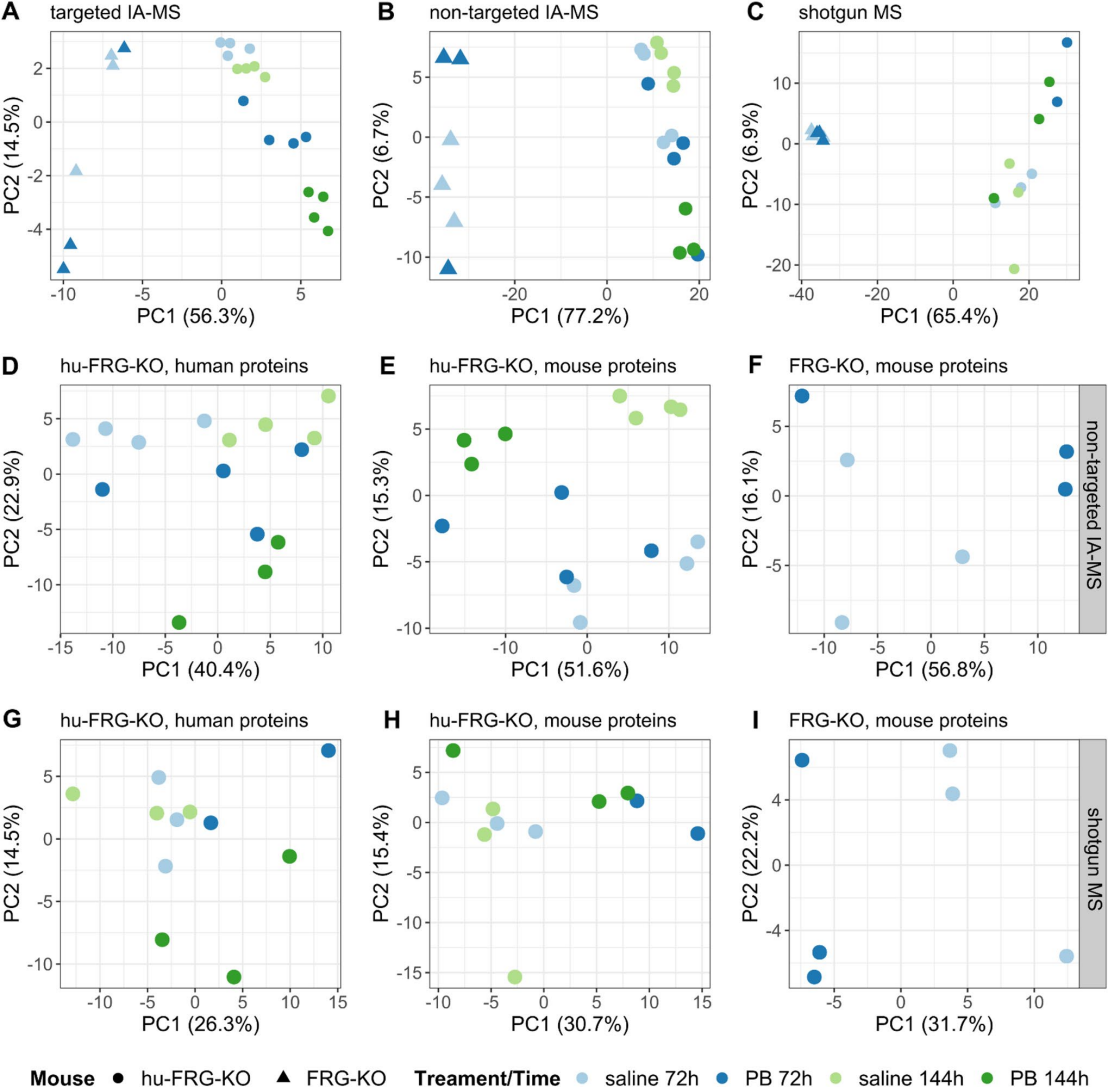
Scores plots of principal component analysis (PCA) for targeted IA-MS (**A**), non-targeted IA-MS (**B**) and shotgun MS (**C**). Colors indicate the different treatments: saline 72 h (light blue), PB 72 h (dark blue), saline 144 h (light green), PB 144 h (dark green), and symbols indicate mouse types (circle, hu-FRG-KO; triangle, FRG-KO). Scores plots of PCA for sample subsets from non-targeted are shown for IA-MS (**D**–**F**) and shotgun MS (**G**–**I**)

PCA was applied to visualize and compare the different data sets. Mouse type was the major factor influencing the variation in all three data sets (Fig. 2A–C), as samples from humanized mice were clearly separated from the other samples by PC1 explaining most of the variation. For a more detailed inspection of the treatment effect, each mouse type and protein type (human/mouse) was analyzed separately for the non-targeted IA-MS and shotgun MS data sets (Fig. 2D–I). This means that both, human and mouse proteins, were individually analyzed from samples of hu-FRG-KO mice, where a small fraction of remnant murine hepatocytes and the non-parenchymal cell fraction contribute to the occurrence of mouse proteins in the humanized livers. One sample (ABE-7) from a hu-FRG-KO mouse strongly deviated from the others in terms of the fractions of human and mouse proteins detected (Supplemental Figure S3). This sample was, therefore, removed from the data set prior to further analysis.

PCA score plots of human proteins in hu-FRG-KO samples visualized a clear effect of PB treatment for both methods, especially after 144 h (Fig. 2D, G). A similar picture could be drawn for mouse proteins in hu-FRG-KO samples; however, this was less evident for the shotgun MS data (Fig. 2E, H). The score plots of samples from non-repopulated FRG-KO mice indicated an evident treatment effect, especially for the shotgun MS data (Fig. 2F, I).

To investigate the PB treatment effect on different mouse types, we performed *t*-tests for each non-targeted data set separately (Table 2). The absolute and relative numbers of significantly changed proteins were largely similar for both methods, non-targeted IA-MS and shotgun MS. There was a slightly higher number of regulated mouse proteins in FRG-KO mice found in the shotgun MS data set, which can most likely be explained by the overall higher number of proteins detected by this method. Finally, we combined the significantly altered proteins of the two complementary data sets. Overall, there was a tendency that more proteins were differentially regulated after longer PB treatment.

**Table 2:** Summary of statistical analysis for PB treatment effects in mice after 72 and 144 h (significance cutoff *p* < 0.05)

Method/Species	Conditions	hu-FRG-KO		FRG-KO
		72 h	144 h	72 h
Targeted IA-MS	Human	9 (31.0%)	16 (55.2%)	–
	Mouse	5 (21.7%)	8 (34.8%)	3 (13.0%)
Non-targeted IA-MS	Human	24 (16.0%)	50 (33.0%)	–
	Mouse	22 (10.5%)	136 (64.5%)	11 (5.2%)
Shotgun MS	Human	67 (6.9%)	55 (5.6%)	–
	Mouse	80 (16.0%)	5 (1%)	112 (8.4%)
Non-targeted methods combined	Human	89	102	–
		**72 ↑ 17 ↓**	**64 ↑ 38 ↓**	
	Mouse	101	141	121
		**15 ↑ 86 ↓**	**12 ↑ 129 ↓**	**77 ↑ 44 ↓**

The percentage is referring to the ratio of significantly affected proteins to all detected proteins per condition

### Targeted IA-MS: PB effects on CYP and transport protein expression

As a starting point of interpretation of the proteomics data, we were interested whether the characteristic model response to CAR activation, namely the induction of xenobiotic-metabolizing enzymes, had been achieved in the experiment. To this end, we investigated the protein levels of CYPs and transport proteins in detail, by applying targeted IA-MS assays. In total, 18 human- and 14 mouse-specific proteins were detected, while the other proteins detected by the assays were not specific for one species.

**Fig. 3:**
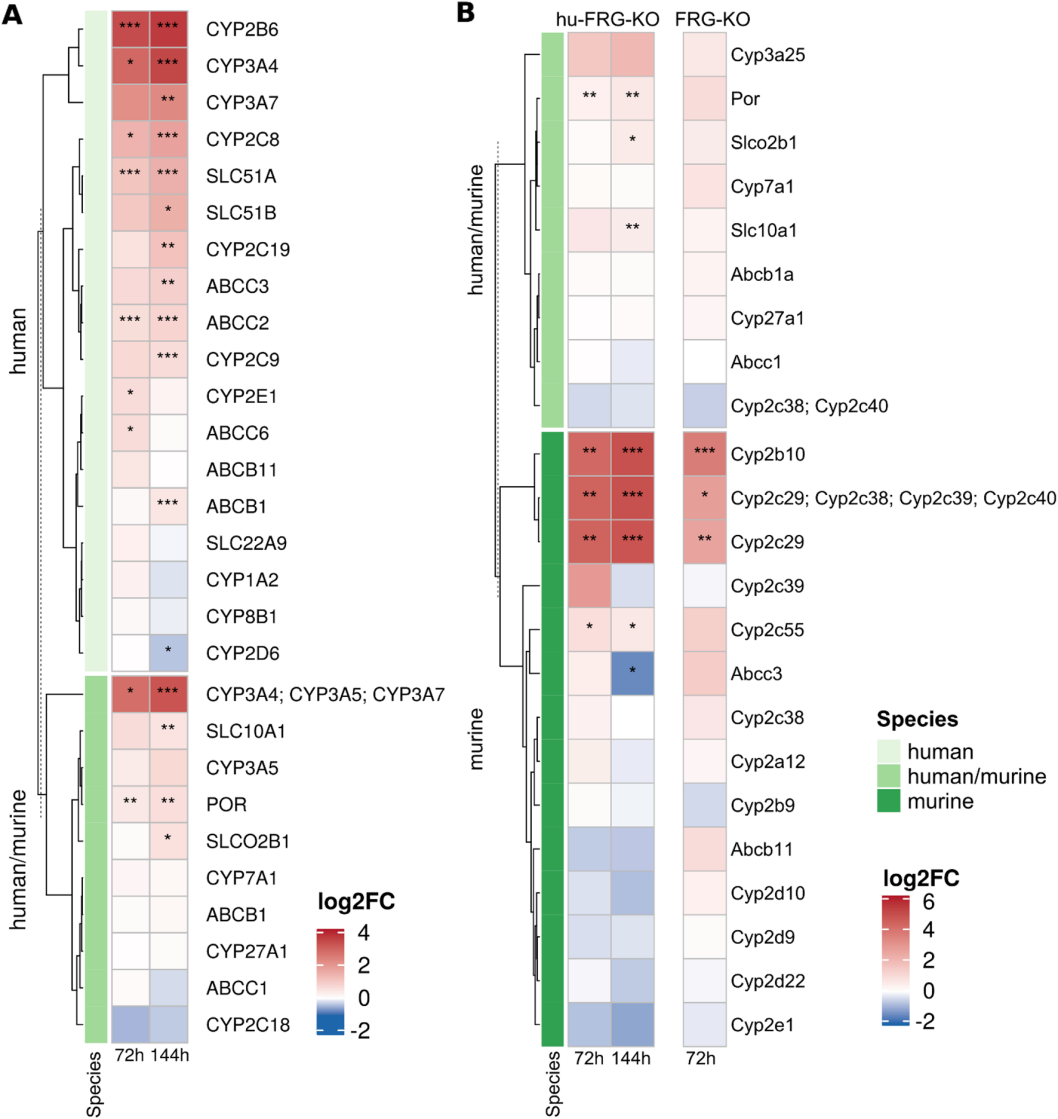
Heatmap showing log_2_ fold change values for the targeted IA-MS data set comprising CYPs and transport proteins. Data for human (**A**) and mouse proteins (**B**) are provided separately (*n* = 3–4). Statistical significance: ***p < 0.001, **p < 0.01, *p < 0.05

The PCA scores plot of the targeted IA-MS data (Fig. 2A) shows a clear clustering of samples by mouse type and PB treatment, especially for hu-FRG-KO mice. This observation was reflected by the high proportion of detected proteins which showed significantly changed levels following PB treatment PB (Table 2; Fig. 3). The strongest response was observed for hu-FRG-KO mice with 9 and 16 significantly changed proteins after 72 and 144 h, respectively. Especially, the proteins CYP2B6, CYP3A4, CYP2C8, ABCC2, POR and SLC51A accumulated upon PB treatment. The response in FRG-KO mice was similar but somewhat less pronounced, where mainly Cyp2b10, several Cyp2c isoforms, Cyp2a12 and Por were up-regulated (Fig. 3; Supplemental Table S3). Thus, in summary, the data demonstrate the PB-dependent up-regulation of known CAR targets related to xenobiotic metabolism, such as murine Cyp2b10 or human CYP2B6. An overall similar picture was observed for human and mouse cells.

### Non-targeted MS: Complementarity and bioinformatics analysis

Comparison of the significantly regulated proteins identified in hu-FRG-KO mice by the two different non-targeted methods (shotgun MS and IA-MS) showed that there was a rather limited overlap, whereas most of the significantly regulated proteins were exclusively detected by one method (Supplemental Figure S4). Only four human proteins (POR, CES1, AKR1B10 and CYP2B6) were found significantly up-regulated by both methods per time point (Supplemental Figure 4A), and one mouse protein (Tpmt) was found consistently down-regulated (Supplemental Figure 4B). Of note, CYP2B6 and POR had also been measured by targeted IA-MS, where they had shown a statistically significant accumulation upon treatment at both time points (Fig. 3), consistent with the results from the non-targeted approaches. Irrespective of the complementarity of data sets, the concordance of results obtained by individual methods should be emphasized, as visualized for CYP proteins in Supplemental Figure S5, thus underlining the validity of the obtained results.

**Fig. 4:**
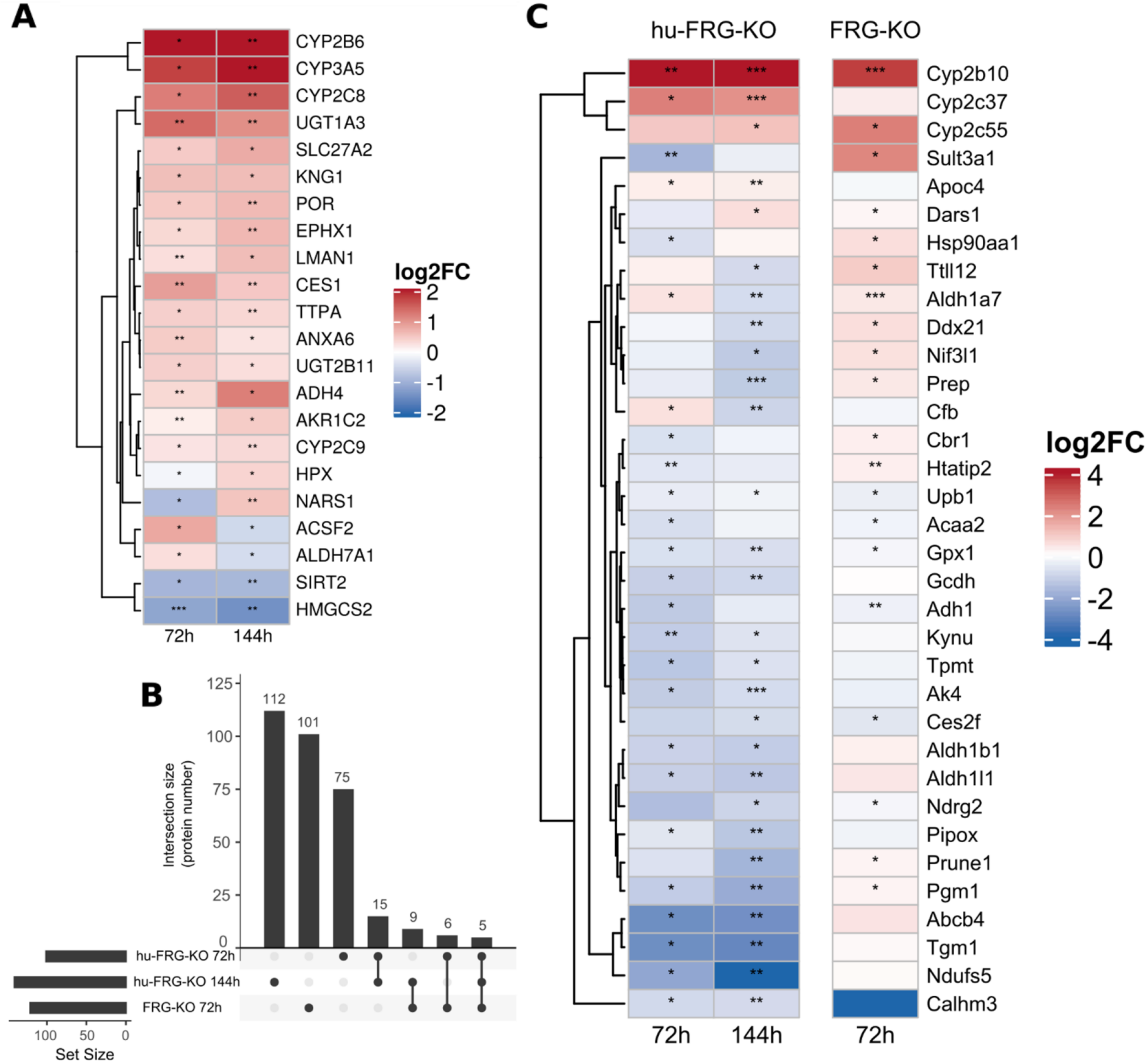
Significantly regulated proteins for different conditions using combined results of the two untargeted methods. **A** Heatmap of log_2_ fold changes for 22 commonly regulated human proteins in hu-FRG-KO (significant after 72 and 144 h). **B** UpSet plot comparing significantly regulated mouse proteins for hu-FRG-KO mice repopulated with human hepatocytes and FRG-KO mice without donor after 72 and 144 h. **C** Heatmap of log_2_ fold changes for 34 differentially regulated mouse proteins. Proteins with significant changes in at least two conditions (*n* = 3–4 mice per condition) are shown and were filtered for NAs (not available). ***p < 0.001, **p < 0.01, *p < 0.05

For further biological interpretation of the results, we combined the significantly regulated proteins found by at least one non-targeted method. No merging with targeted data was performed, as data resulting from the latter approach were specifically focused on drug and xenobiotic metabolism, and thus not expected to remarkably contribute to the elucidation of additional biological functions affected by PB. Supplemental Figure 6 shows that approx. 20 proteins per class (human proteins, mouse proteins in hu-FRG-KO mice) were significantly regulated at both time points, i.e., after 72 h as well as after 144 h. While most of these proteins showed the same direction of change relative to control conditions at both time points, there were a few exceptions. In case of the human proteins, four (HPX, NARS1, ACSF2, ALDH7A1) out of 22 overlapping proteins reflected this observation (Fig. 4). The majority of proteins, however, showed the same direction and often a slightly higher extent of change after 144 h, e.g., CYP3A5, EPHX1, ADH4 and HMGCS2 (Fig. 4A). A similar trend was visible for mouse proteins significantly regulated in hu-FRG-KO mice (Fig. 4C). Comparing both mouse types, we observed an opposite trend of regulation for numerous proteins, especially after 144 h of PB treatment (Fig. 4C). A much higher number of mouse proteins was strongly down-regulated after 144 h in humanized mice, as compared to the situation for murine proteins after 72 h in hu-FRG-KO or in FRG-KO mice. The overall response of mouse hepatocytes in hu-FRG-KO and FRG-KO mice at the 72 h time point was very similar (Fig. 4C).

**Fig. 5:**
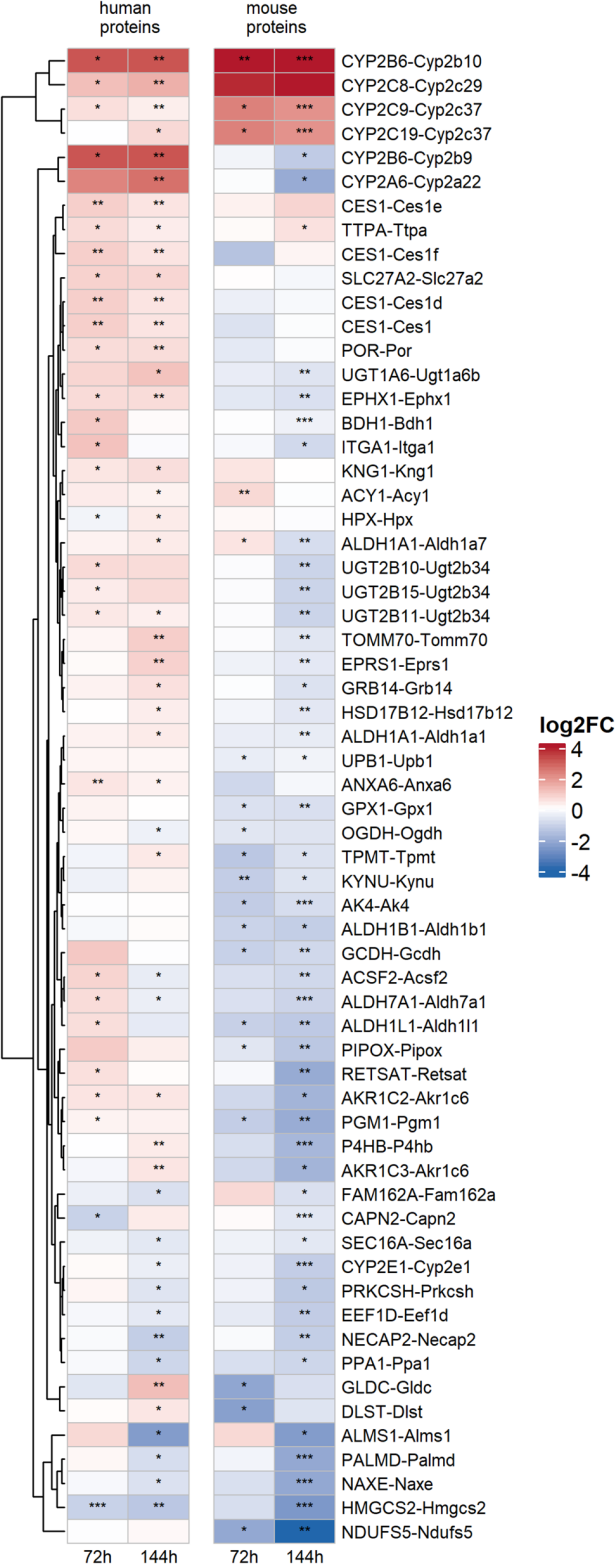
Heatmap of log_2_ fold changes for significantly regulated proteins in hu-FRG-KO mice using combined results from shotgun MS and non-targeted IA-MS. Human and mouse proteins were matched by ortholog information (described in “Materials and methods”). Proteins with significant changes in at least two conditions (*n* = 3–4 mice per condition) are shown and were filtered for NAs (not available). ***p < 0.001, **p < 0.01, *p < 0.05

To allow for comparison of human and mouse proteins that were measured in samples of hu-FRG-KO mice, we used ortholog information from HCOP to link them (see “Materials and methods”). Figure 5 depicts the log_2_ fold changes for a subset of significantly regulated human and mouse proteins reflecting different patterns of PB treatment (a more extensive heatmap including additional proteins can be found in Supplemental Figure S7). There is a small number of up-regulated human proteins (CYP2B6, CYP2C8, CYP2C9 and CYP2C19) where the ortholog mouse proteins (Cyp2b10, Cyp2c29 and Cyp2c37) showed the same trend. However, we observed the opposite effect for another group of human proteins that were up-regulated (CYP2B6, CYP2A6), whereas their mouse orthologs were down-regulated (Cyp2b9, Cyp2a22). Of note, the same tendency had been observed for CYP2B6-Cyp2b9 measured by targeted IA-MS (Fig. 3). There were several more cases fitting this pattern (especially after 144 h treatment), e.g., EPRS1-Eprs1, TOMM70-Tomm70, ALDHA1-Aldha1, EPHX1-Ephx1, UGT1A6-Ugt1a6b, TPMT-Tpmt, AKR1C2-Akr1c6 and P4HB-P4hb. Finally, there were some protein pairs where the human and the mouse orthologs were down-regulated after 144 h of PB treatment, e.g., PALMD-Palmd, CYP2E1-Cyp2e1, ALDH7A1-Aldh7a1, ALMS1-Alms1 and HMGCS2-Hmgcs2. Overall, this comparison shows that human and mouse protein networks reflect common PB effects but also numerous differences.

### Functional enrichment analysis

**Fig. 6:**
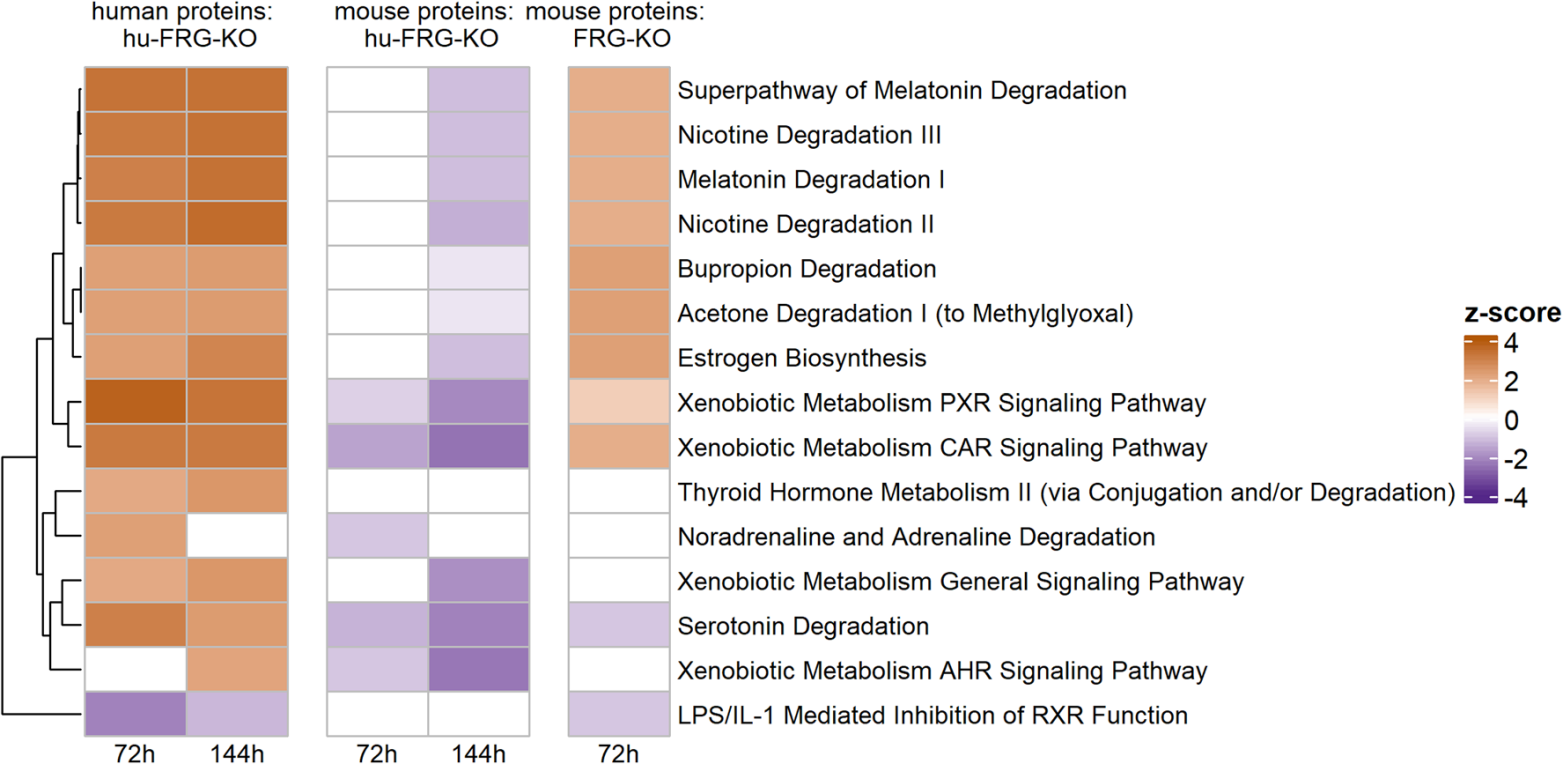
IPA Canonical pathway analysis of combined data. Only species-specific proteins were analyzed for each mouse type and time point. Values of absolute *z*-score > 2 should be regarded significant. Only pathways that were significant for at least one group of mice were included in the heatmap

We performed a functional evaluation of the regulated proteins using different levels of annotation within Ingenuity Pathway Analysis (IPA). Based on the combined results of significant protein changes, IPA canonical pathway analysis indicated a strong activation of xenobiotic metabolism, nicotine degradation and estrogen biosynthesis for human proteins in hu-FRG-KO mice and for mouse proteins in FRG-KO mice (Fig. 6). However, an opposite trend was observed for mouse proteins in hu-FRG-KO mice, mainly after 144 h of PB treatment.

**Fig. 7:**
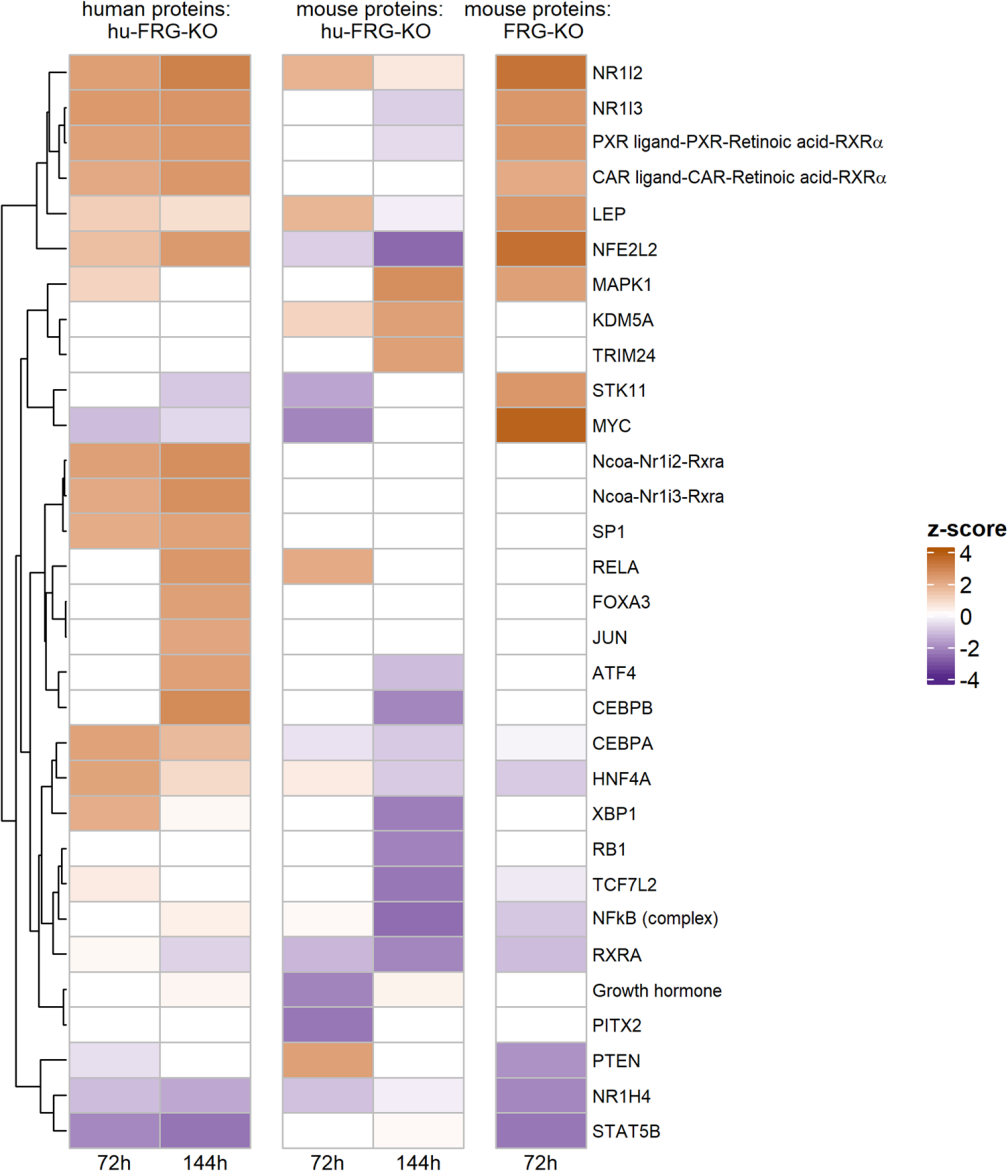
IPA Upstream regulator analysis of combined data. Only species-specific proteins were analyzed for each mouse type and time point. Values of absolute *z*-score > 2 should be regarded significant. Only pathways that were significant for at least one group of mice were included in the heatmap

The results for IPA upstream regulator analysis are depicted in Fig. 7. For human proteins in hu-FRG-KO mice, the top activated regulators were CAR (NR1I2), PXR (NR1I3), NFE2L2 (NRF2), CEBPB, and RELA, while NR1H4 and STAT5B were predicated as inactivated. This reflects the induction of xenobiotic metabolism. A similar trend was observed for mouse proteins in FRG-KO mice, where also CAR, PXR, and NRF2 activation was prominent. However, some particular differences were observed: here, a number of regulators was predicted as activated, which are linked to cell proliferation and/or development cancer. IPA predicted an activation of the mitogen-activated protein kinase 1 (MAPK1), an inactivation of the tumor suppressor PTEN, and, most prominently, strong activation of the proto-oncogene MYC, crucially involved in hepatocellular proliferation following CAR activation (Blanco-Bose et al. 2008; Braeuning et al. 2011a). Of note, MYC activation was not predicted for human proteins in hu-FRG-KO mice, where MYC appeared to be slightly down-regulated. This is well in line with the findings from the BrdU incorporation analysis (cp. Fig. 1), thus validating the results of our study with regard to the capacity of PB to induce proliferation in human and mouse hepatocytes.

Besides the functional enrichment analysis of combined data sets, we also investigated the individual datasets in more detail. To assess the effect of selecting only species-specific proteins on the downstream interpretation, we compared them to all proteins including the not species-specific ones. Regarding the non-targeted IA-MS data, the IPA analyses of canonical pathways, upstream regulators and diseases & bio functions were leading to quite similar conclusions for both protein sets, all versus species-specific (Supplemental Figures S8–S10). However, some additional pathways and functions appeared as enriched considering all mouse proteins in hu-FRG-KO mice 144 h after PB treatment, for instance the necroptosis signaling pathway (inactivated), apoptosis (activated) and cell viability (inactivated).

Within the shotgun MS data, we observed overall similar enrichment results for all detected human proteins compared to specific proteins (Supplemental Figures S11–S13). Focusing on mouse proteins, the choice of protein selection makes a difference for the functional interpretation in hu-FRG-KO mice, especially for 72 h after PB treatment. Some enriched pathways (e.g., xenobiotic metabolism, NRF2 mediated oxidative stress response, hepatic steatosis) showed an opposite pattern depending on the protein set.

Interestingly, the upstream regulator MYC emerged in more cases when considering all proteins rather than just species-specific proteins. IPA predicted an inactivation of MYC for hu-FRG-KO mice, but a strong activation for FRG-KO mice (Supplemental Figures S9, S12). This again underlines the striking difference between mouse and human hepatocytes.

## Discussion

The FRG-KO and PXB mouse models share many similarities regarding their general applicability to study the behavior of human hepatocytes in vivo, but differ in the molecular alterations utilized to allow for the repopulation of livers by human hepatocytes. While a detailed presentation of all aspects of genetic differences between the two models would by far exceed the scope of this paper, it should be noted that, as a matter of principle, certain strain-specific findings and/or artifacts might always result from the use of transgenic strains with such severe phenotypes. Therefore, replication of previous results in a second chimeric mouse model is crucial to increase the credibility of data. This is especially relevant in the present case, as besides the difference in their genetic background the mice in the study by Yamada et al. (2014) were supplemented with human growth hormone, and an interaction of growth hormone with phenobarbital-dependent effects cannot be excluded. To this regard, hu-FRG-KO mice constitute a complementary model by using different biology of hepatocyte deficiency, which does not require the use of growth hormone in the humanized animals during the study. Female mice were used, instead of male mice in the Yamada study, because the proliferative response to CAR activation in mice is known to be substantially higher in females than in males (Braeuning et al. 2011b).

Repopulation by human hepatocytes adds a different layer of complexity to the mouse models and might also affect the outcome of a study. In contrast to the study by Yamada and co-workers, where hepatocytes were used from a pediatric donor aged 2 years (Yamada et al. 2014), we have consciously chosen a 13-year-old hepatocyte donor for our analysis. The reason for that was that it has been reported that PB administration starting at very young age of mice, as early as 2 weeks after birth, results in tumor inhibition but not promotion, while applying the same treatment regimen a little later to mice aged 6 weeks, will lead to tumor-promoting effects of PB (e.g., see the review by Lee (2000)). Therefore, it is not clear whether hepatocytes from a too young human donor might affect the outcome of a study on endpoints related to proliferation and/or tumor formation, provided that the abovementioned contradictory responses are depending on the age of the hepatocytes and not on other yet unknown physiological factors.

The present results are nonetheless well in line with previous findings from Yamada and co-workers (Yamada et al. (2020); Yamada et al. (2014)) who showed a clear-cut proliferative response in murine hepatocytes. Of note, a tendency towards a PB-dependent increase in proliferation was visible in human hepatocytes in our study, but did not reach the criteria of statistical significance. Similarly, Yamada et al. (2014) observed a tendency for slightly elevated levels of replicative DNA synthesis in their study as well. One might speculate that the statistically not significant differences, which have been observed twice in studies with only few animals, might reach statistical criteria in a possible follow-up experiment with a remarkably increased number of chimeric mice. Nonetheless, it has to be noted that even in case of reaching statistical significance in another study, it would be expected that the response of human hepatocytes is much less than of mouse hepatocytes. This is further supported by findings in humanized CAR/PXR mice: there, a clear-cut increase in the proliferation marker Ki-67 was reported in hepatocytes from wild-type mice following treatment with the CAR activator cyproconazole, whereas only a non-significant tendency for increased proliferation was observed in the humanized animals (Marx-Stoelting et al. 2017). The small animal numbers are certainly shortcomings to our study as well as to the Yamada study, because practical and financial constraints limit the possibilities when drafting such study protocols.

Another interesting aspect is related to the elevated liver weight in hu-FRG-KO mice. Under conditions of a normal healthy liver, PB treatment induces a moderate liver weight increase. Size of the liver is a tightly regulated process, which is still not fully understood. It appears that PB forces liver growth towards a new steady state. Notably, the relative liver weights of hu-FRG-KO mice not treated with PB were already higher than the weights reached in normal livers with PB treatment (see this work, but also cp. with previously published data, e.g., Braeuning et al. (2011b); Marx-Stoelting et al. (2017)). It, therefore, appears possible that the basal elevated liver weights of the chimeric mice preclude a further hypertrophic and/or hyperplastic growth during PB exposure, as the actual liver size already exceeds what might be reached via PB-mediated CAR activation.

However, a continued debate on the possible proliferative response in human hepatocytes might still not solve all questions related to the issue of PB-mediated tumorigenesis. Tumor cells promoted by PB or similarly acting compounds in the livers of mice bear, in the vast majority of cases, mutations leading to an activation of the canonical Wnt/β-catenin signaling pathway (Aydinlik et al. 2001; Mattu et al. 2018; Strathmann et al. 2006). Amongst having also other functions, permanently activated β-catenin is considered an oncogenic driver in different tumor entities, in mice as well as in humans (He and Tang 2020). The small population of initiated hepatocytes with activated β-catenin are the cells, which are in fact promoted during chronic treatment with PB. Observations whether normal hepatocytes, i.e., with intact signaling but without mutational activation of β-catenin, show transient proliferation or not following exposure to PB, does unfortunately not allow drawing firm conclusions for the putative behavior of β-catenin-activated cells exposed to PB. Therefore, analyses of the transient proliferation response of normal hepatocytes are by definition not suited to prove the relevance or non-relevance of CAR-dependent tumor promotion, in any species.

Nonetheless, with regard to the issue of induction of proliferation as a consequence of CAR activation, the present study underlines that marked differences exist between mouse and human hepatocytes. Our study constitutes an important extension of previous knowledge on humanized mouse livers and their response to xenobiotics, as for the first time we have analyzed the response of human hepatocytes in vivo at a proteomic level. A novel workflow was established to analyze proteins from two species within one sample, and a highly valuable data set was assembled by combining complementary targeted and non-targeted approaches.

## Supplementary Information

Below is the link to the electronic supplementary material.

Supplementary file1 (DOCX 1554 KB)

Supplementary file2 (XLSX 1494 KB)

## Abbreviations

CAR: Constitutive androstane receptor
CYP: Cytochrome P450
LLOQ: Lower limit of quantification
TXP: Triple X proteomics
IA-MS: Immunoaffinity mass spectrometry
PB: Phenobarbital
PCA: Principal component analysis
