# Supplementary material for: Proteomic analysis of hepatic effects of phenobarbital in mice with humanized liver
Source: Arch Toxicol. 2022 Jul 26;96(10):2739–54. doi: 10.1007/s00204-022-03338-7 (PMC9352639; doi:10.1007/s00204-022-03338-7)
Supplement: Supplementary file 1 — Supplementary file1 (DOCX 1554 KB) [file 204_2022_3338_MOESM1_ESM.docx]

**Supplemental Material**

**Proteomic Analysis of Hepatic Effects of Phenobarbital in Mice with Humanized Liver**

Heike Sprenger^1^, Josef Daniel Rasinger^2^, Helen Hammer^3^, Wael Naboulsi^3^, Elke Zabinsky^4^, Hannes Planatscher^3^, Michael Schwarz^4^, Oliver Poetz^3,5^, Albert Braeuning^1*^

^1^German Federal Institute for Risk Assessment, Dept. Food Safety, Berlin, Germany

^2^Institute of Marine Research (IMR), Postboks 1870 Nordnes, NO-5817 Bergen, Norway

^3^SIGNATOPE GmbH, Markwiesenstr. 55, 72770 Reutlingen, Germany

^4^University of Tübingen, Dept. of Experimental and Clinical Pharmacology and Pharmogenomics, Wilhelmstr. 56, 72074 Tübingen, Germany

^5^NMI Natural and Medical Sciences Institute at the University of Tuebingen, Markwiesenstr. 55, 72770 Reutlingen, Germany

*Corresponding author: Albert Braeuning, German Federal Institute for Risk Assessment, Dept. Food Safety, Max-Dohrn-Str. 8-10, 10589 Berlin, Germany, Phone +49-(0)30-18412-25100, Fax +49-(0)30-18412-99099, E-mail: Albert.Braeuning@bfr.bund.de

**Supplemental Tables**

**Table S1.** Liver and body weight data of mice at sample harvest. The sample removed from shotgun MS analysis as an outlier is indicated in yellow color in the table.

| **Mouse ID** | **Donor** | **Group Name** | **Mouse Type** | **Treatment** | **Time** | **Body Weight (g)** | **Liver (g)** | **Liver/Body Weight (%)** |
| --- | --- | --- | --- | --- | --- | --- | --- | --- |
| 64187 | HHF13023 | hu-FRG KO | humanized | 0.9% Saline | 72 h | 21.5 | 2.36 | 10.98 |
| 64194 | HHF13023 | hu-FRG KO | humanized | 0.9% Saline | 72 h | 24.4 | 2.23 | 9.14 |
| 64369 | HHF13023 | hu-FRG-KO | humanized | 0.9% Saline | 72 h | 23.0 | 2.47 | 10.74 |
| 64197 | HHF13023 | hu-FRG-KO | humanized | 0.9% Saline | 72 h | 21.6 | 2.17 | 10.05 |
| 64243 | HHF13023 | hu-FRG-KO | humanized | 50 mg/kg PB | 72 h | 24 | 2.66 | 11.08 |
| 64245 | HHF13023 | hu-FRG-KO | humanized | 50 mg/kg PB | 72 h | 25.6 | 3.02 | 11.80 |
| 64246 | HHF13023 | hu-FRG-KO | humanized | 50 mg/kg PB | 72 h | 19.6 | 2.06 | 10.51 |
| 64249 | HHF13023 | hu-FRG-KO | humanized | 50 mg/kg PB | 72 h | 22.7 | 2.35 | 10.35 |
| 64198 | HHF13023 | hu-FRG-KO | humanized | 0.9% Saline | 144 h | 21.8 | 2.23 | 10.23 |
| 64201 | HHF13023 | hu-FRG-KO | humanized | 0.9% Saline | 144 h | 18.1 | 1.73 | 9.56 |
| 64203 | HHF13023 | hu-FRG-KO | humanized | 0.9% Saline | 144 h | 19.8 | 2.07 | 10.45 |
| 64204 | HHF13023 | hu-FRG-KO | humanized | 0.9% Saline | 144 h | 24.4 | 2.39 | 9.80 |
| 64347 | HHF13023 | hu-FRG-KO | humanized | 50 mg/kg PB | 144 h | 18.1 | 1.9 | 10.50 |
| 64350 | HHF13023 | hu-FRG-KO | humanized | 50 mg/kg PB | 144 h | 27.8 | 2.73 | 9.82 |
| 64351 | HHF13023 | hu-FRG-KO | humanized | 50 mg/kg PB | 144 h | 25.1 | 2.64 | 10.52 |
| 64358 | HHF13023 | hu-FRG-KO | humanized | 50 mg/kg PB | 144 h | 20.3 | 2.37 | 11.67 |
| 65960 | NA | FRG-KO | without donor | 0.9% Saline | 72 h | 30 | 1.59 | 5.30 |
| 65961 | NA | FRG-KO | without donor | 0.9% Saline | 72 h | 28.8 | 1.62 | 5.63 |
| 65965 | NA | FRG-KO | without donor | 0.9% Saline | 72 h | 28.1 | 1.47 | 5.23 |
| 65975 | NA | FRG-KO | without donor | 50 mg/kg PB | 72 h | 34 | 2.17 | 6.38 |
| 65976 | NA | FRG-KO | without donor | 50 mg/kg PB | 72 h | 21.2 | 1.78 | 8.40 |
| 65977 | NA | FRG-KO | without donor | 50 mg/kg PB | 72 h | 33.1 | 2.01 | 6.07 |

**Table S2.** Statistical summary of phenotypic data of mice at sample harvest

**Table S3.** Analyzed proteins are listed with uniprot ID and surrogate peptide used for the quantification by targeted IA-MS

**Table S4.** Targeted IA-MS data set including 48 proteins that correspond to 42 unique analytes (also considering the species specificity)

**Table S5**. Non-targeted immunoaffinity proteomics (IA-MS) data set comprising of 1032 peptides corresponding to 819 unique proteins

**Table S6**. Shotgun MS data set including 2913 unique proteins that were quantified by species-specific peptides. Filtering of the data set was performed as described in Materials and Methods

**Supplemental Figures**

**
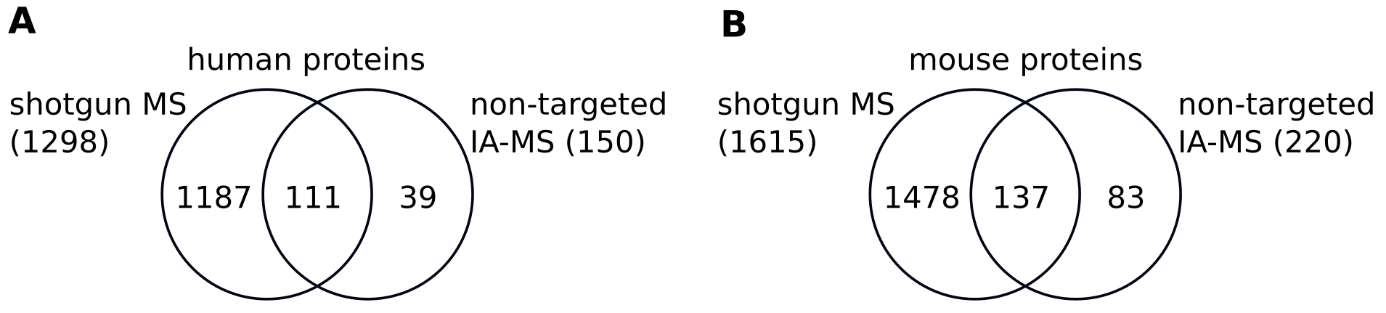
**

Figure S1. Overlap of proteins detected by shotgun MS and non-targeted IA-MS.


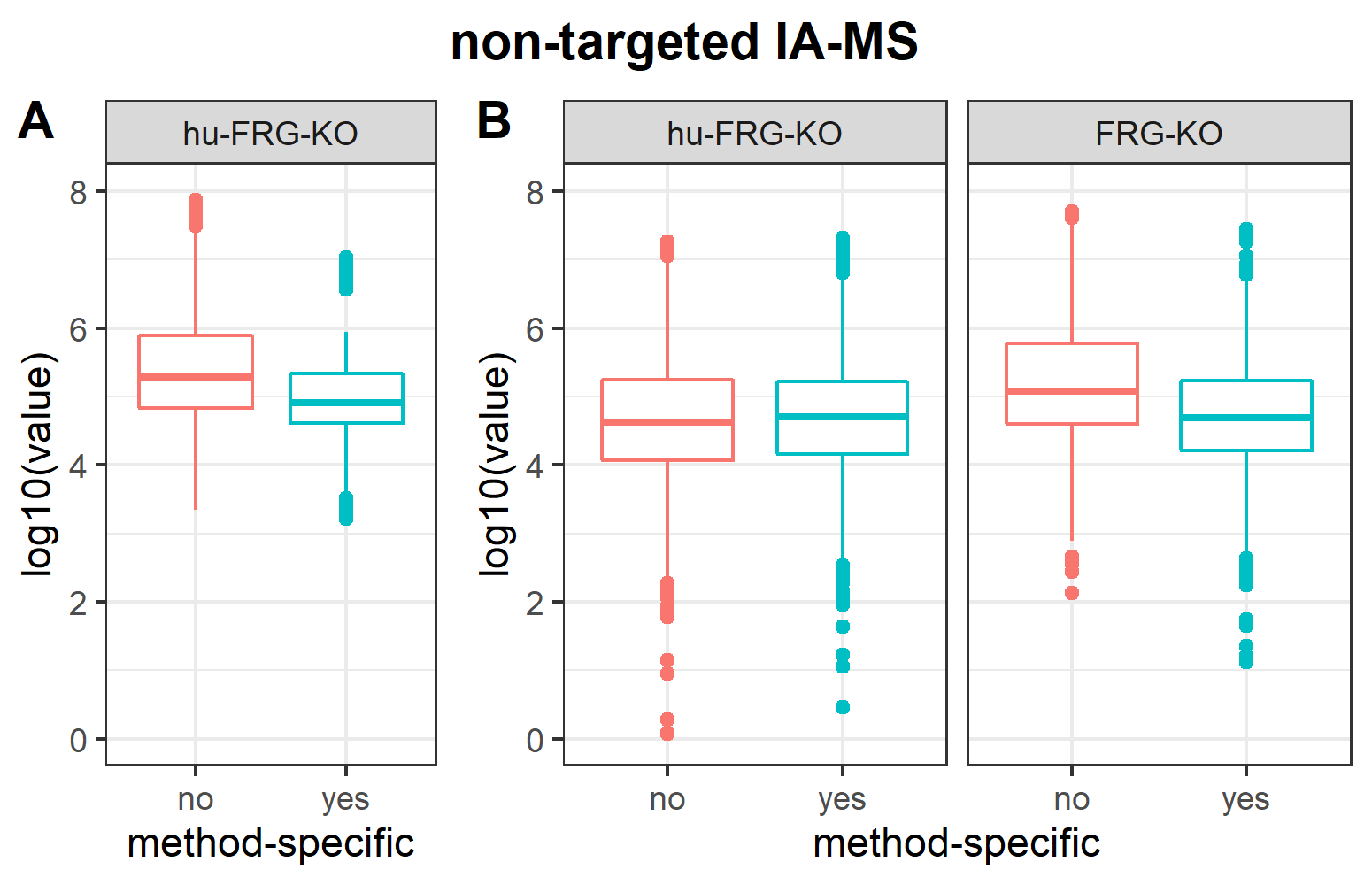

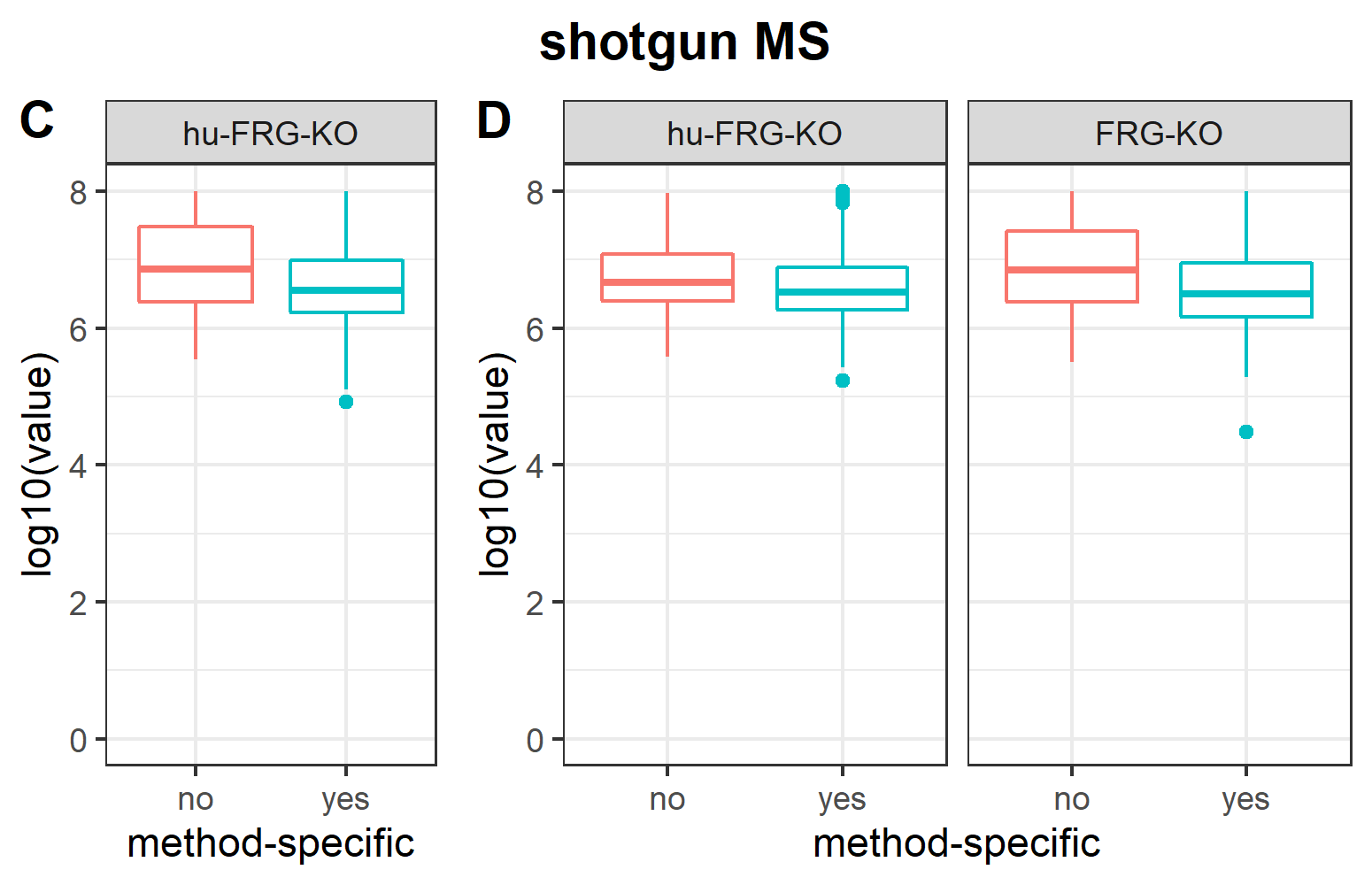


Figure S2. Comparison of intensity for non-targeted IA-MS data and shotgun MS data. Proteins that were only detected by non-targeted IA-MS or by both methods (non-targeted IA-MS and shotgun MS) are shown per mouse type for human proteins (A) and mouse proteins (B). Proteins that were only detected by shotgun MS or by both methods (non-targeted IA-MS and shotgun MS) are shown per mouse type for human proteins (C) and mouse proteins (D).


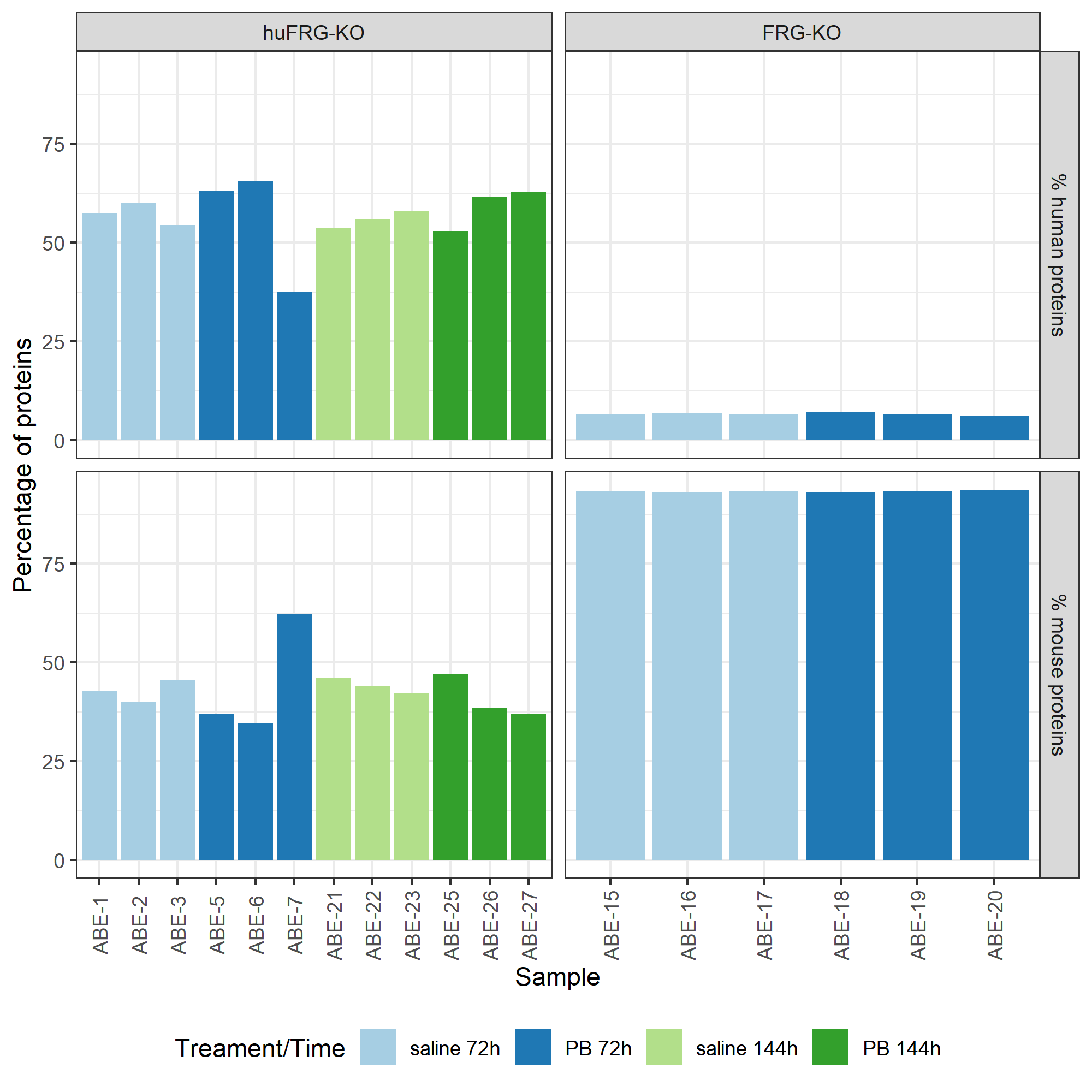


Figure S3. Percentage of proteins detected per sample relative to the total number of human/mouse proteins.


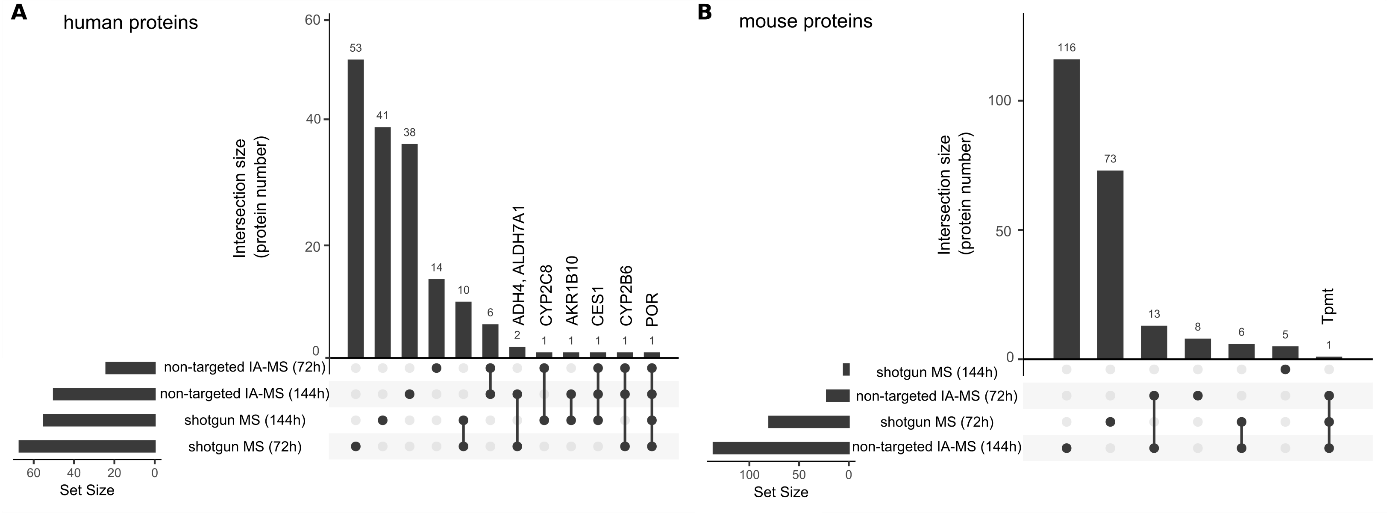


Figure S4. UpSet plot comparing the significantly regulated proteins using shotgun MS or non-targeted IA-MS for human proteins (A) and mouse proteins (B) in hu-FRG-KO mice.


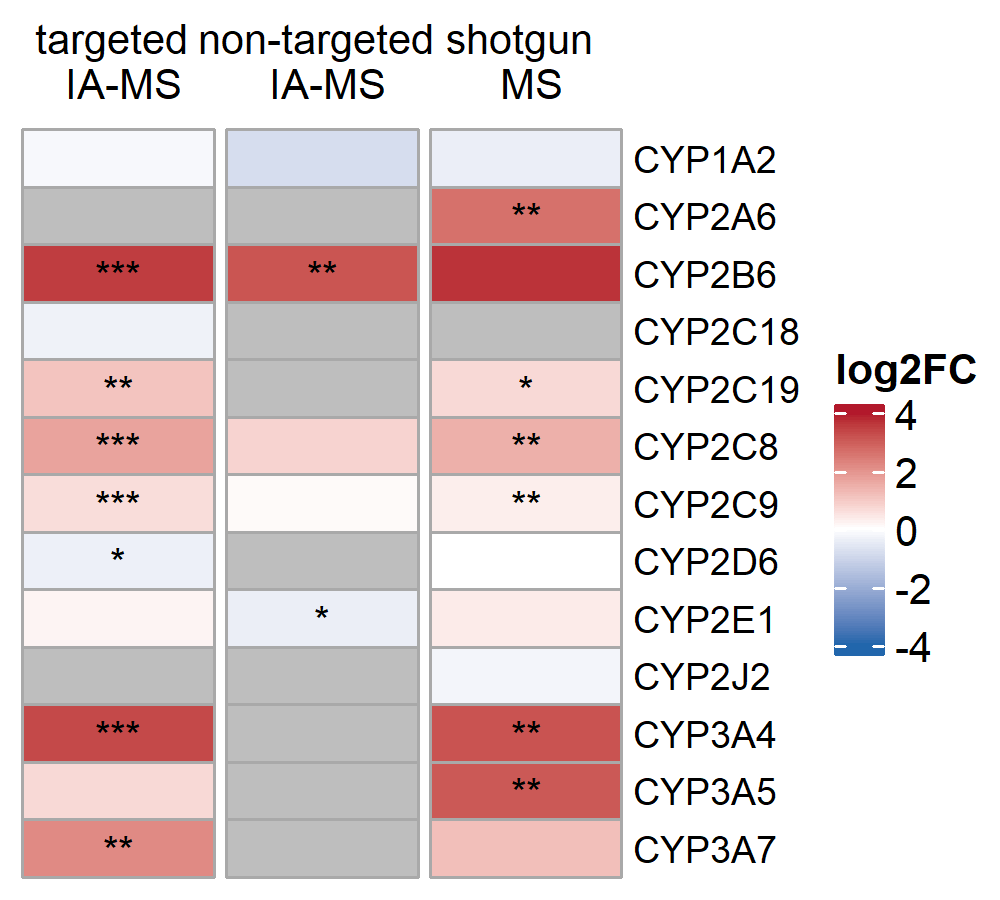


Figure S5. Concordance of results obtained by different proteomic techniques. Findings obtained for xenobiotic-metabolizing CYPs from families 1-3 are shown. Up- or downregulation is indicated by color; gray: not determined. Proteins are significantly changed are indicated as follows: *** p < 0.001, ** p < 0.01, * p < 0.05.­


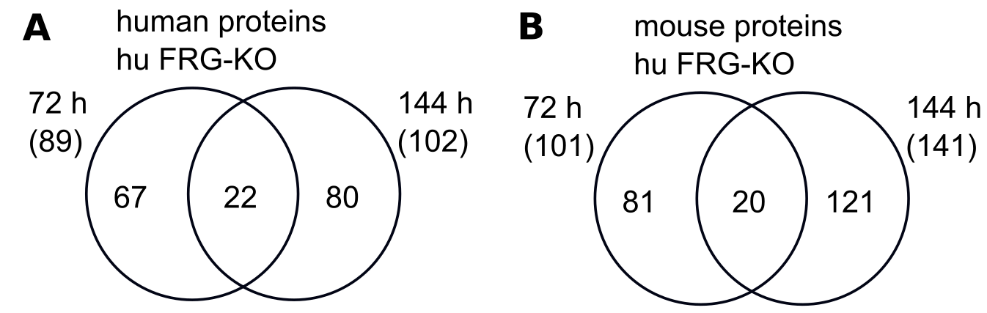


Figure S6. Overlap of significantly regulated proteins of PB treatment effect after 72 and 144 hours per mouse type (using combined results of two methods).


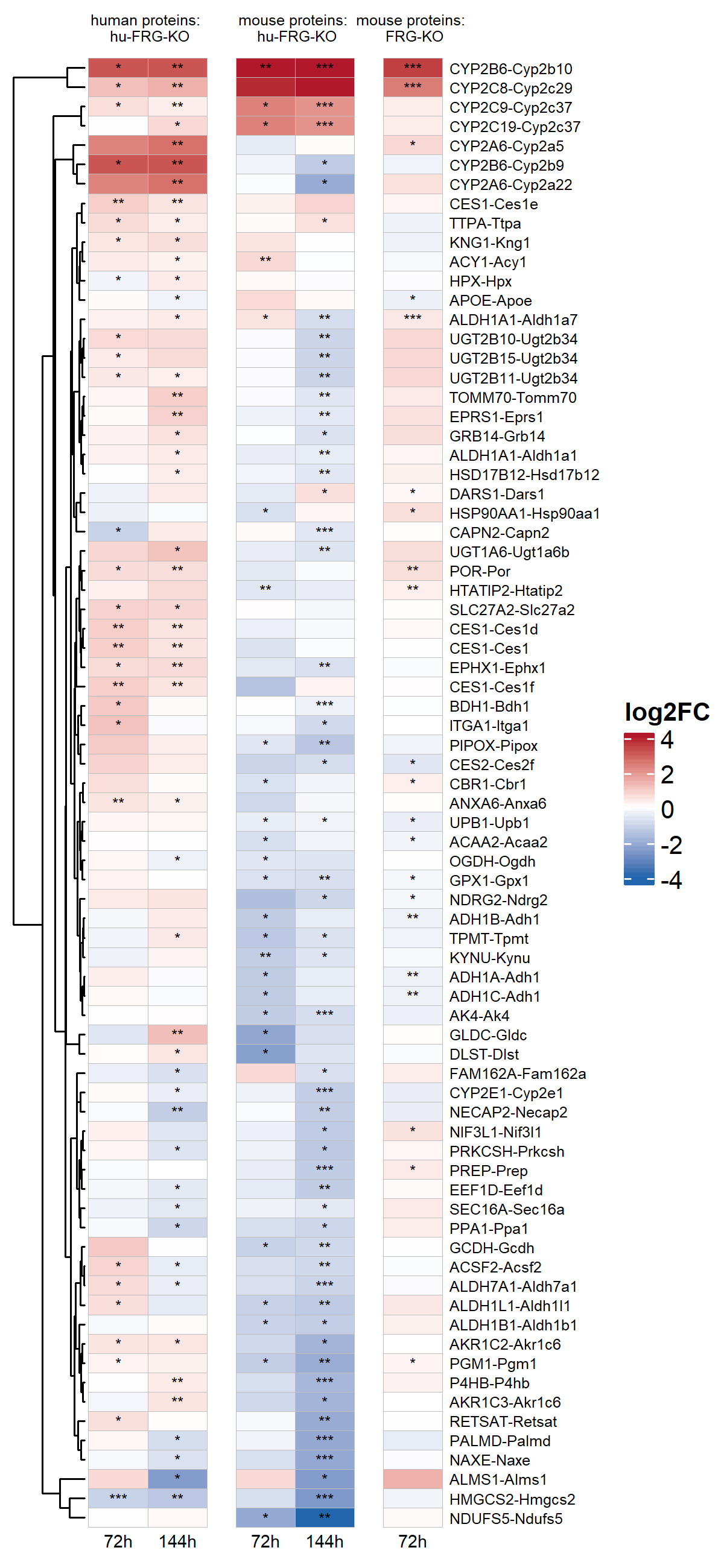


Figure S7. Heatmap of log_2_ fold changes for significantly regulated proteins in hu-FRG-KO and FRG-KO mice using combined results. Human and mouse proteins were matched by ortholog information (described in Materials & Methods). Proteins are significantly changed in at least two conditions and are filtered for NAs (not available). *** p < 0.001, ** p < 0.01, * p < 0.05.­


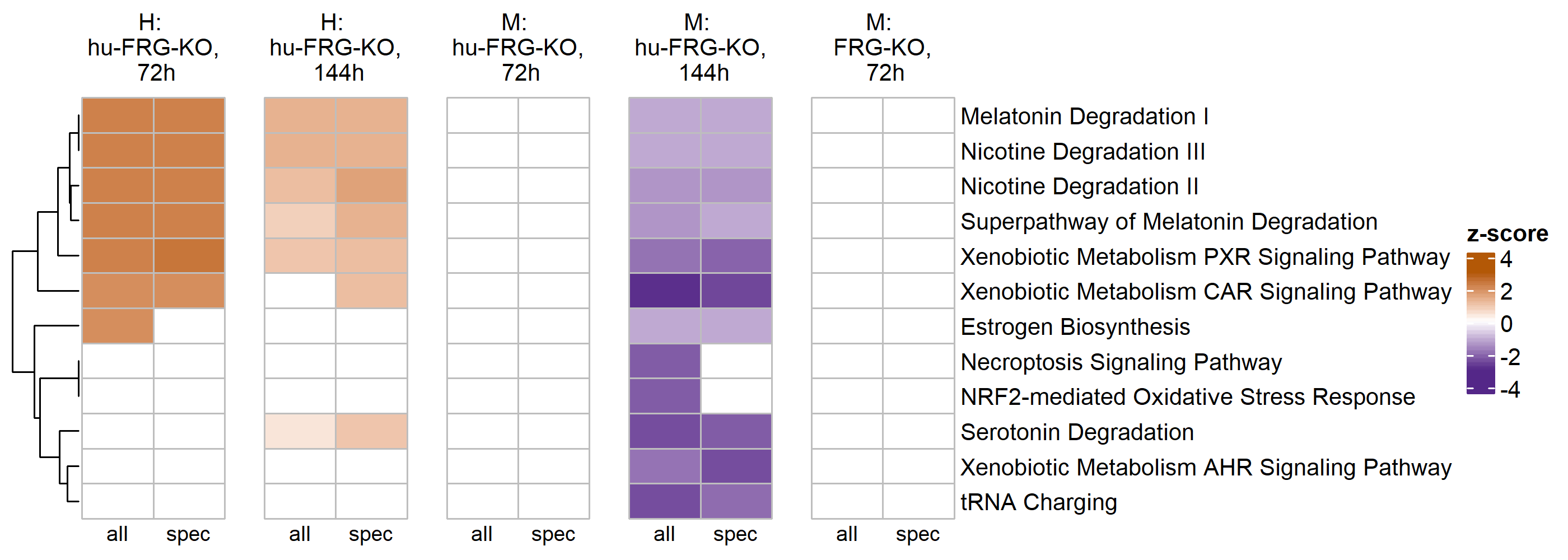


Figure S8. IPA Canonical pathway analysis of non-targeted IA-MS data comparing all proteins vs. species-specific proteins. Values of absolute z-score > 2 should be regarded significant, only pathways that were significant for at least one case were included in the heatmap. The letters H and M indicate human and mouse proteins, respectively.


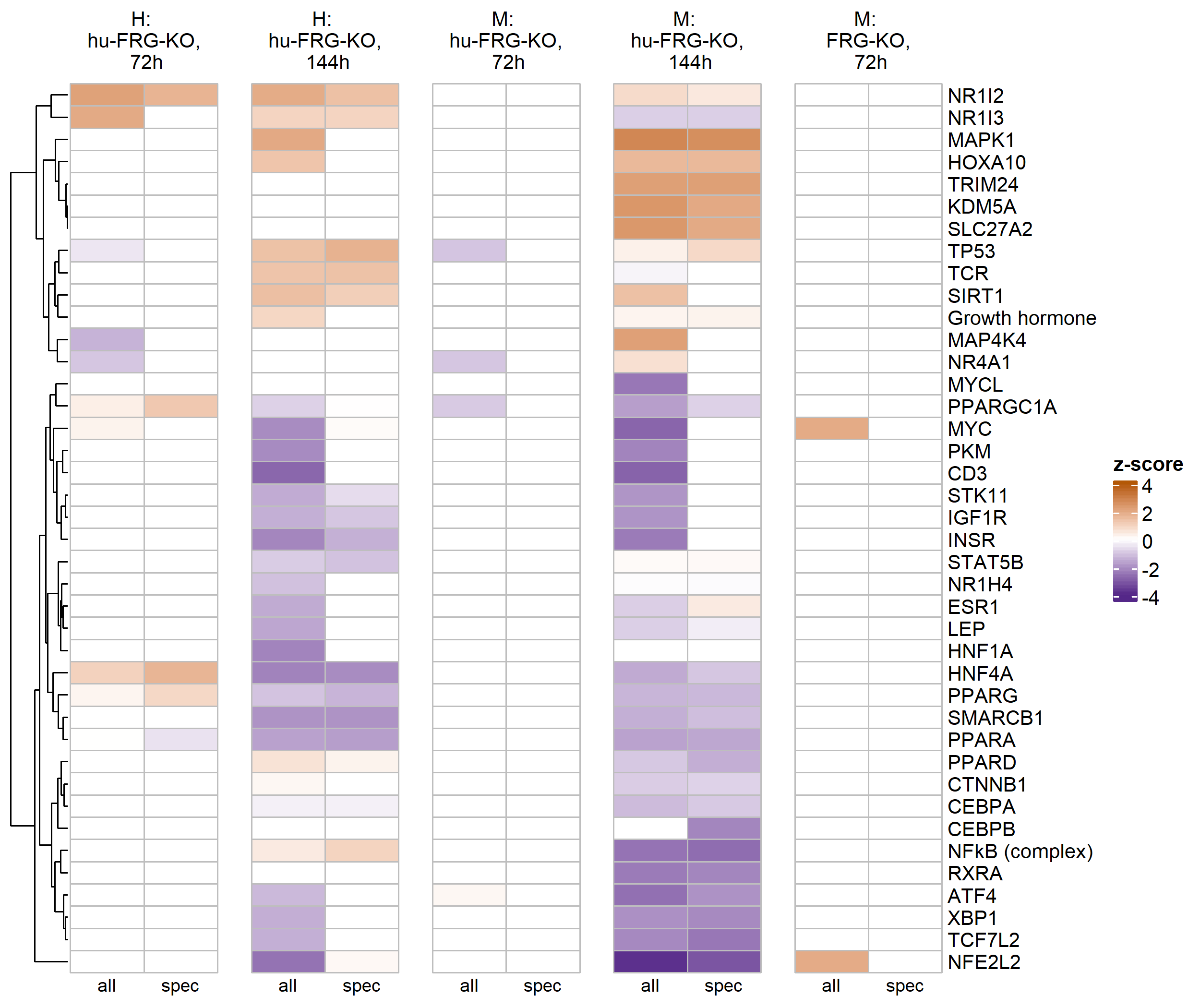


Figure S9. IPA Upstream regulators analysis of non-targeted IA-MS data comparing all proteins vs. species-specific proteins. Values of absolute z-score > 2 should be regarded significant, only regulators that were significant for at least one case were included in the heatmap. The letters H and M indicate human and mouse proteins, respectively.


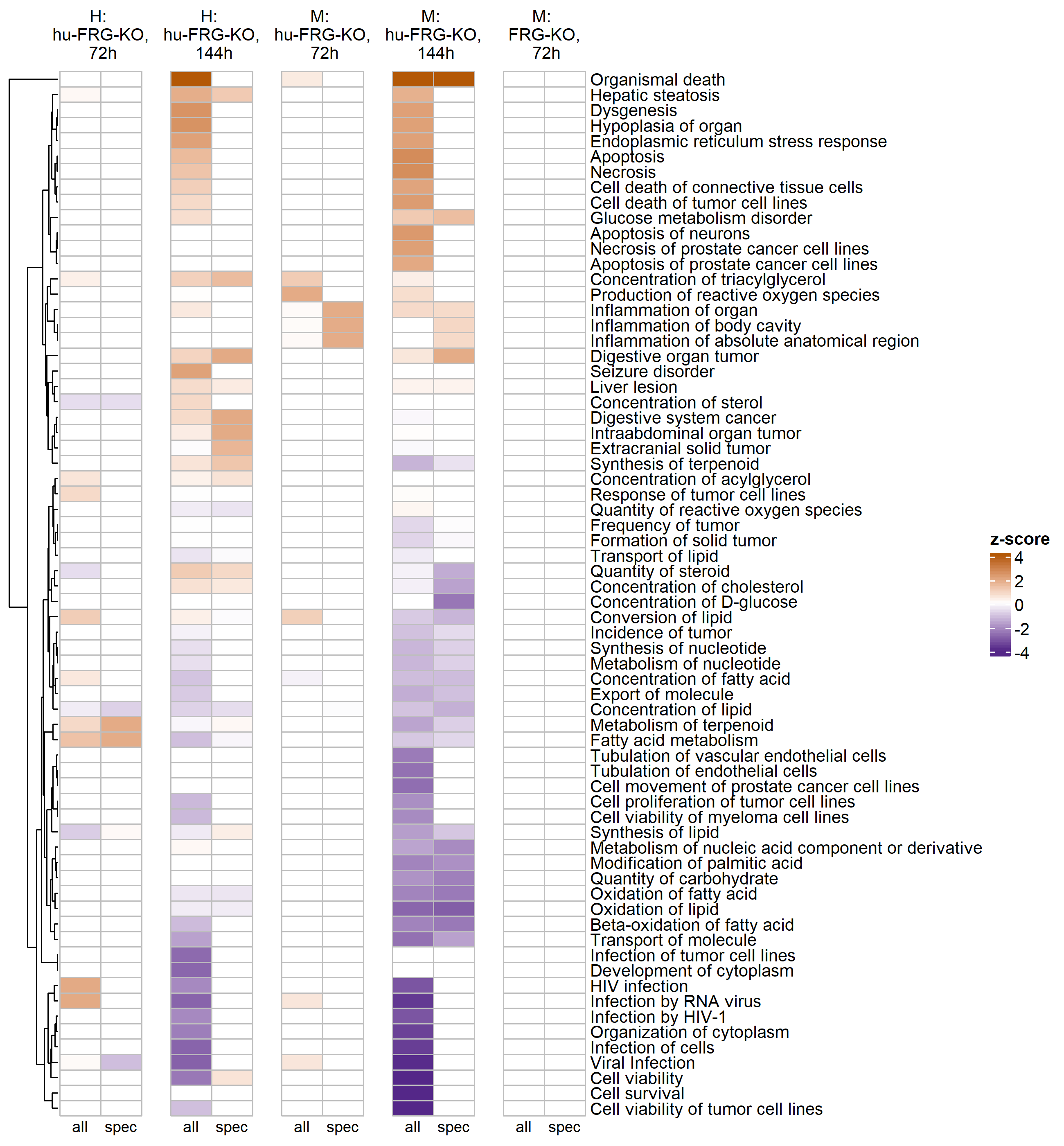


Figure S10. IPA Diseases & bio functions analysis of non-targeted IA-MS data comparing all proteins vs. species-specific proteins. Values of absolute z-score > 2 should be regarded significant, only categories that were significant for at least one case were included in the heatmap. The letters H and M indicate human and mouse proteins, respectively.


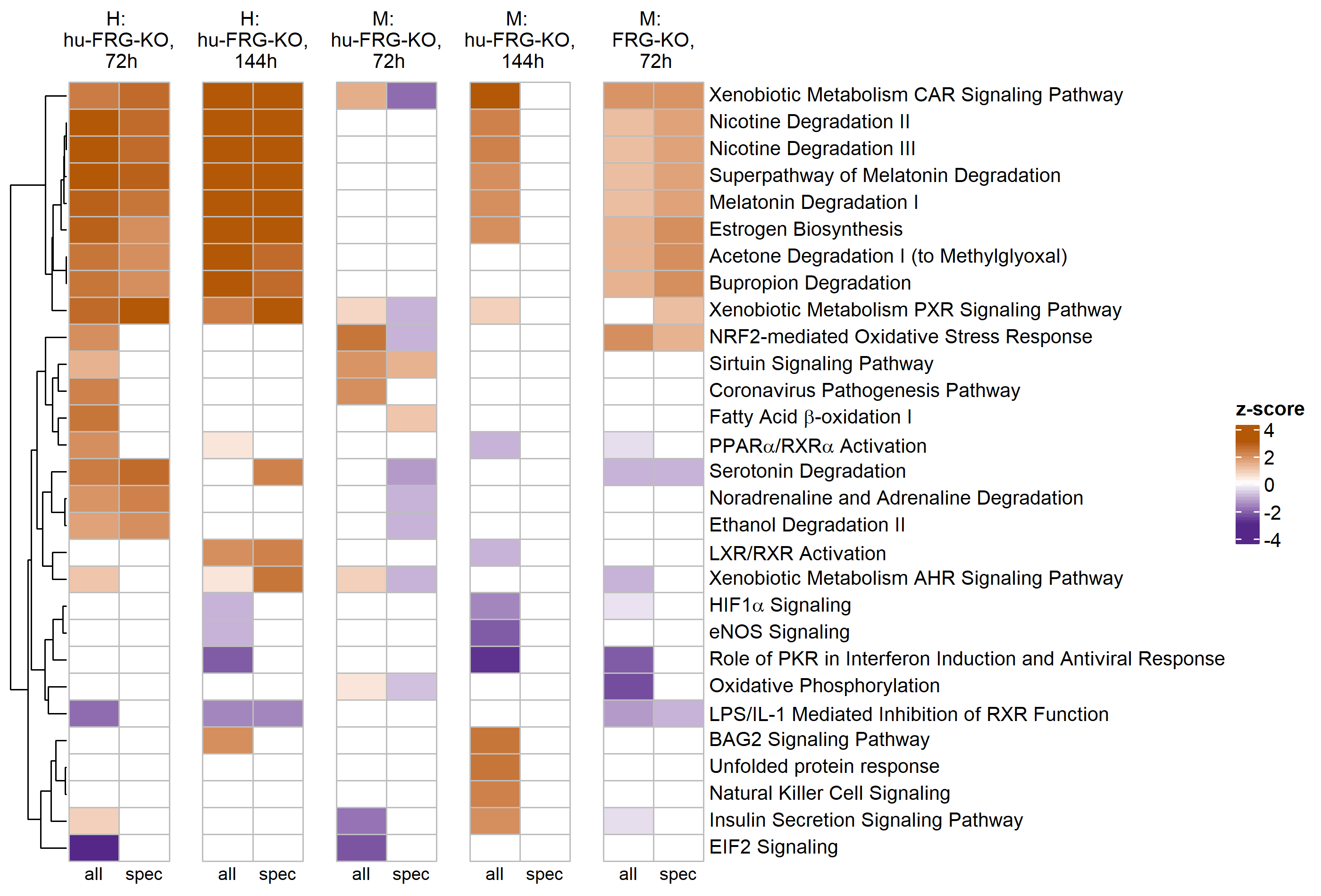


Figure S11. IPA Canonical pathway analysis of shotgun MS data comparing all proteins vs. species-specific proteins Values of absolute z-score > 2 should be regarded significant, only pathways that were significant for at least one case were included in the heatmap.. The letters H and M indicate human and mouse proteins, respectively.


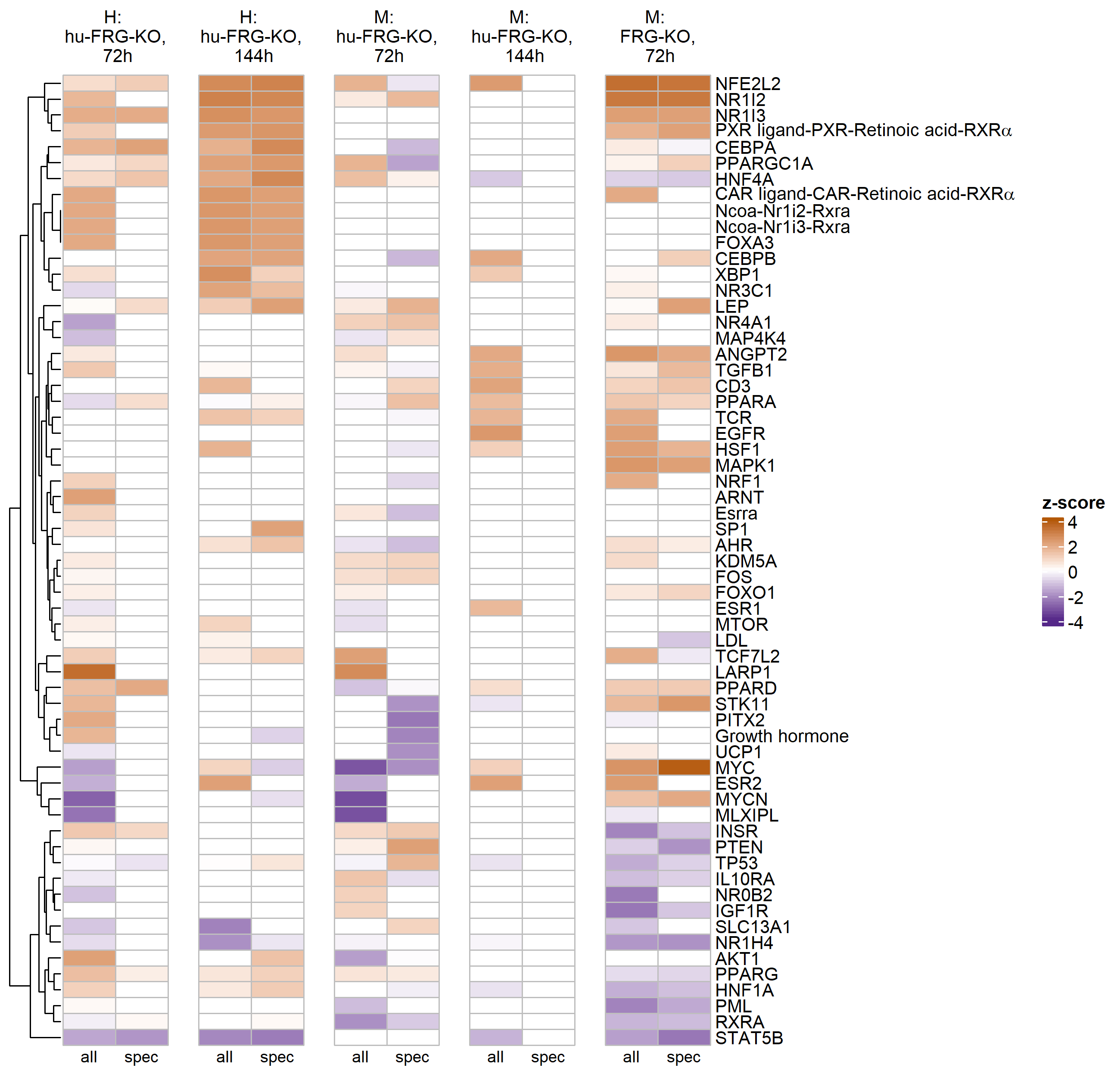


Figure S12. IPA Upstream regulators analysis of shotgun MS data comparing all proteins vs. species-specific proteins. Values of absolute z-score > 2 should be regarded significant, only regulators that were significant for at least one case were included in the heatmap. The letters H and M indicate human and mouse proteins, respectively.


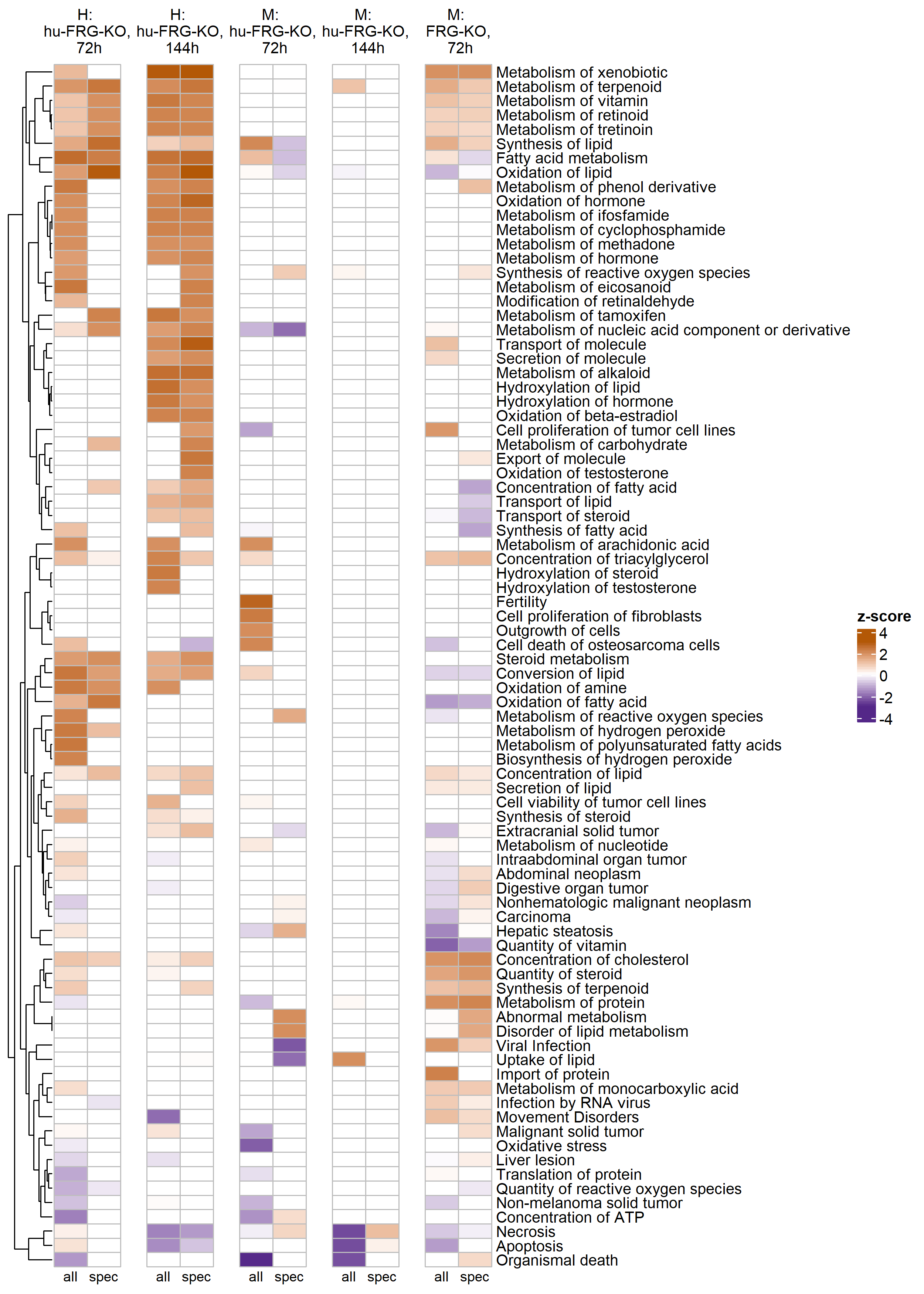


Figure S13. IPA Diseases & bio functions analysis of shotgun MS data comparing all proteins vs. species-specific proteins. Values of absolute z-score > 2 should be regarded significant, only categories that were significant for at least one case were included in the heatmap. The letters H and M indicate human and mouse proteins, respectively.
